# Pentafluoroorthotellurate Uncovered: Theoretical Perspectives on an Extremely Electronegative Group

**DOI:** 10.1021/acs.inorgchem.4c04603

**Published:** 2025-01-03

**Authors:** Daniel Barrena-Espés, Ángel Martín Pendás, Sebastian Riedel, Alberto Pérez-Bitrián, Julen Munárriz

**Affiliations:** † Departamento de Química Física y Analítica, Universidad de Oviedo, Oviedo 33006, Spain; ‡ Fachbereich Biologie, Chemie, Pharmazie, Institut für Chemie und Biochemie − Anorganische Chemie, Freie Universität Berlin, Fabeckstraße 34/36, Berlin 14195, Germany; § Institut für Chemie, Humboldt-Universität zu Berlin, Berlin 12489, Germany; ∥ Departamento de Química Física and Instituto de Biocomputación y Física de Sistemas Complejos (BIFI), Universidad de Zaragoza, Zaragoza 50009, Spain

## Abstract

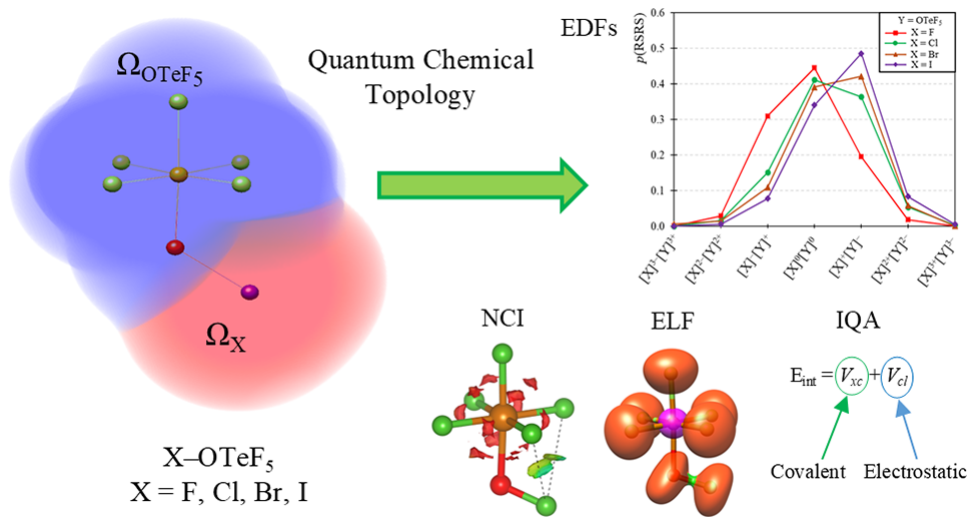

The pentafluoroorthotellurate group (−OTeF_5_, teflate) exhibits high electron-withdrawing properties. Indeed, it is often used as a bulky substitute for fluoride due to its high chemical stability and larger size, which reduces its tendency to act as a bridging ligand. These characteristics make it a valuable ligand in synthetic chemistry, facilitating the preparation of molecular structures analogous to polymeric fluoride-based compounds. In this study, we explore the electronic structure of the teflate group by using advanced Quantum Chemical Topology (QCT) methods to better understand its bonding nature and compare its group electronegativity with that of the halogens. For that, we examine XOTeF_5_ systems (X = F, Cl, Br, I) and decompose X–OTeF_5_ interactions into classical (ionic) and exchange-correlation (covalent) contributions by using interacting quantum atoms (IQA) energy decomposition scheme. We also conduct a detailed analysis of electron distribution by utilizing the statistical framework of electron distribution functions (EDFs) and examine the electron localization function (ELF), electron density, and reduced density gradient scalar functions, as well as delocalization indices and QTAIM charges. The results show that the electron-withdrawing properties of the teflate group are comparable to those of fluorine, albeit slightly lower. Moreover, its internal bonding is primarily ionic. Additionally, we compare −OTeF_5_ with other O-donor groups, demonstrating that the electron-withdrawing properties within OEF_5_ (E = S, Se, Te) systems are nearly identical, and these groups show a higher group electronegativity than OCF_3_, OC(CF_3_)_3_, and OC_6_F_5_.

## Abstract 2

We compare −OTeF_5_ with other O-donor groups, demonstrating that electronegativity within OEF_5_ (E = S, Se, Te) systems is nearly identical, and these groups show a higher group electronegativity than OCF_3_, OC(CF_3_)_3_, and OC_6_F_5_.

## Introduction

**Chart 1:**
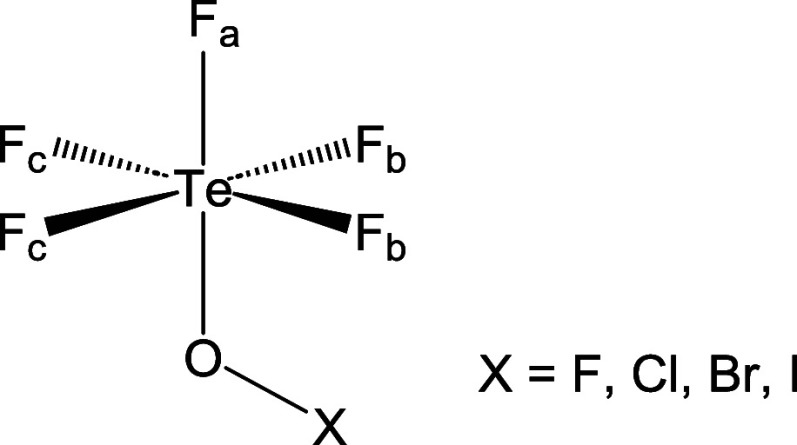
Structure of the Pentafluoroorthotellurate Group within the XOTeF_5_ Systems Considered in This Work (X = F, Cl, Br, I)

The pentafluoroorthotellurate group (−OTeF_5_, teflate, Chart 1) is considered as a bulky analogue of fluoride, with similar electron-withdrawing properties and a high chemical robustness, yet with less tendency to act as a bridging ligand. In fact, it usually forms the only stable analogues of the related fluorides,^1,2^ including compounds in high oxidation states (e.g., [Mo(OTeF_5_)_6_]) or weakly coordinating anions (e.g., [Al(OTeF_5_)_4_]^−^).^3^ The similar electron withdrawing properties of both ligands have been historically assessed in terms of the most stable molecular structures based on the valence shell electron pair repulsion (VSEPR) theory – where the most electronegative group typically occupies a particular position −,^4,5^ Mössbauer quadrupole splittings,^6^ NMR chemical shifts,^5,6^ and vibrational spectroscopy.^4^ Recently, the use of quantum-chemical methods in the [Co(OTeF_5_)_4_]^2–^ system has also demonstrated computationally that fluoride and teflate form Co–X bonds (X = F, OTeF_5_) of comparable strength and lead to a similar charges at the Co^II^ center,^7^ a result that was afterward reproduced for related Mn^II^ and Mn^III^-based systems.^8^ The electronic structure of the teflate anion itself has been previously investigated, although to a much lower level of theory (using Hartree–Fock methodology).^9^ Therefore, we consider that deeper studies using the more accurate and insightful computational methodologies available nowadays are highly desired, and it is our aim to apply them to attain a comprehensive understanding of the teflate group and its analogy with fluoride. This way, a clearer picture and understanding of the above-mentioned properties of the teflate group can also help spread its use in fundamental and applied research. This group offers two key advantages compared to fluoride analogues stemming from its tendency to form species with lower nuclearity. First, it enables the creation of Lewis superacids or weakly coordinating anions with properties superior to those of fluoride analogues. Second, it allows for the formation of coordination complexes that facilitate the study of spectroscopic properties, which are often hindered by the extended structures of related fluoride species.

For this aim, we resorted to the quantum chemical topology (QCT) framework. QCT encompasses a set of methodologies that allow for the partitioning and characterization of molecules and their properties through the topological analysis of various scalar fields derived from the system’s wave function.^10,11^ Arguably, one of the main attractive features of QCT methodologies is the fact that they are invariant under orbital transformations, making them very robust against variations in the level of theory considered.^12^ They are also well-known for their ability to connect quantum mechanics to chemically sound concepts in real space – such as atomic charges, electronegativities and bonding indices – without sacrificing physical rigor.^13^ In this regard, the most celebrated method developed under the QCT umbrella is the quantum theory of atoms in molecules (QTAIM), proposed by Bader. QTAIM is based on the analysis of the topology of the electron density^14^ and is routinely used to characterize chemical bonding.^15^

Nonetheless, other important QCT techniques are gaining significant recognition within the scientific community for their accuracy and chemical insightfulness.^13,16^ In this regard, the interacting quantum atoms (IQA) energy decomposition scheme^17^ allows for a rigorous partition of the chemical space based on the topology of a scalar field, generally, but not limited to, the electron density within the QTAIM formalism. In short, it enables the calculation of the interaction energy between pairs of atoms (or functional groups). Furthermore, it partitions the interaction energy into a classical interaction term (*V*_cl_), which is directly associated with the electrostatic contribution of the interaction, and an exchange-correlation term (*V*_xc_), related to electron-sharing interactions and considered as the covalent counterpart.^18^ This approach has been extensively applied to the understanding of noncovalent interactions, like hydrogen^19^ and halogen bonds,^20^ and metal–metal^21^ and metal–ligand interactions,^22^ among many others.^23^ In this line, it is worth noting that we have recently applied IQA to the study of homoleptic Co and Mn teflate anionic complexes.^7,8^

Electron distribution functions (EDFs) are also of great interest.^24^ Within his approach, given an externally defined partition of a system with *N* electrons into a set of nonoverlapping regions (Ω_1_,···, Ω_m_), one can determine the probability of finding *n*_1_ electrons in Ω_1_, *n*_2_ in Ω_2_, etc. The complete set of all these probabilities is the so-called electron distribution function. The Ω domains can be provided by the QTAIM topology, allowing for the definition of atoms and/or functional groups.^25−27^ Each possible distribution of the system’s *N* electrons across the regions is referred to as a real space resonance structure (RSRS). This way, the system can be described as a set of RSRSs with different probabilities (weights). RSRSs can be directly correlated with Pauling structures, providing an orbital-independent analogue to traditional resonance structures, and revealing important and chemical intuitive information on relevant concepts like electronegativity and bonding character.^28^ Indeed, a system characterized with a broad RSRSs distribution is expected to be rather covalent, as electrons are shared between different centers. On the contrary, if it displays sharp distributions (that is, a high probability associated with a given RSRS) with non-neutral charge maximum-probability RSRS, it indicates highly polar regimes, where certain atoms or functional groups exhibit a significantly increased tendency to attract and *host* electrons.^25^

**Figure 1:**
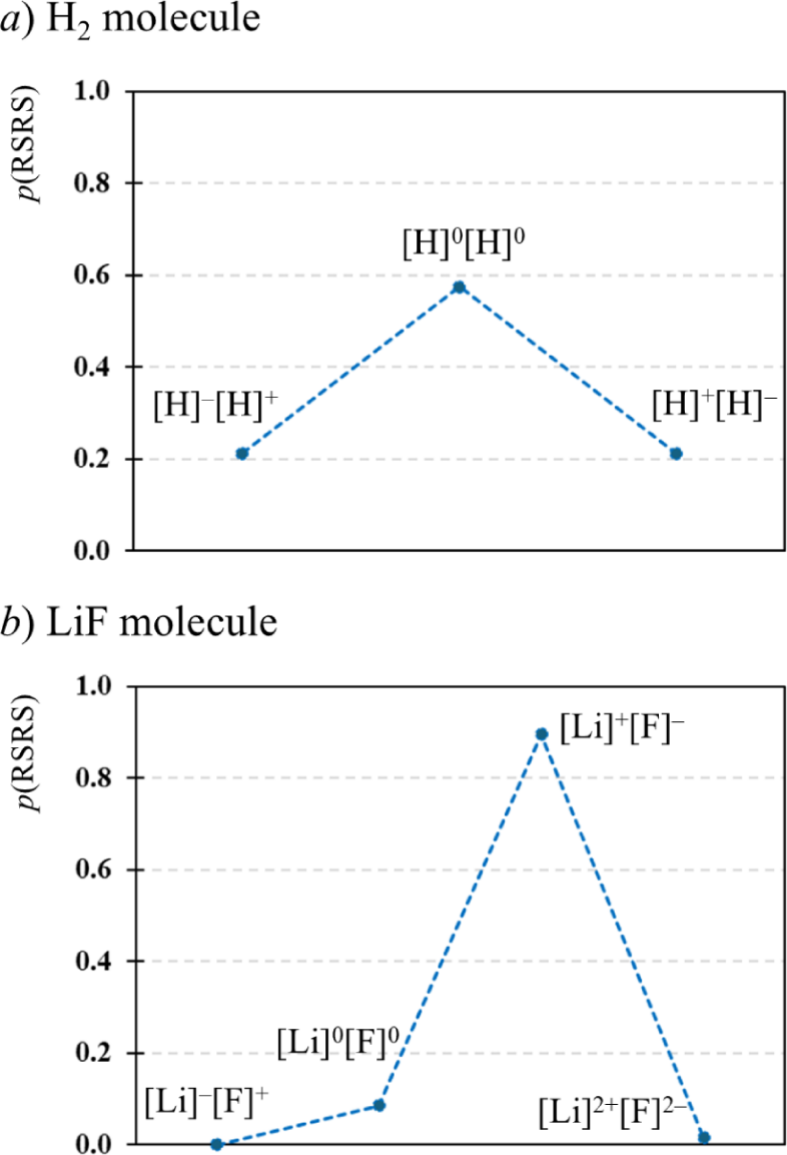
EDF analysis for (a) H_2_ and (b) LiF. Calculations were performed at the CAS(2,2) and CAS(8,5) levels of theory, respectively, using def2-TZVP basis set in both cases.

To clarify the meaning of EDFs to nonspecialists, let us consider two different molecules as an example: LiF and H_2_. The former is considered an ionic system, while the latter is a paradigmatic example of a covalent bonding regime. In H_2_ (2 electrons and 2 atoms: H_A_ and H_B_) there are three possible RSRSs: [H]^0^[H]^0^ (one electron in each atom), [H]^−^[H]^+^ (both electrons in H_A_) and [H]^+^[H]^−^ (both electrons in H_B_). As shown in Figure 1, the probability peak corresponds to [H]^0^[H]^0^, while the probability of [H]^−^[H]^+^ and [H]^+^[H]^−^ is equal and significantly lower, albeit relevant. The symmetrical distribution of H_2_, with a peak at the *neutral* RSRS – [H]^0^[H]^0^ – is indicative of a covalent bonding regime. On the contrary, for LiF (12 electrons and 2 atoms) a single RSRS, [Li]^+^[F]^−^ (2 electrons in Li and 10 electrons in F), nearly represents 90% of the electron distribution population. The following RSRS in relevance is [Li]^0^[F]^0^ (3 electrons in Li and 9 electron in F), with a much lower probability. The asymmetrical distribution of LiF, with a peak at [Li]^+^[F]^−^, a very small weight of [Li]^0^[F]^0^ and no significant probability of [Li]^−^[F]^+^ is indicative of a very polar (or ionic) bonding regime.

Overall, it should be noted that the statistical interpretation of chemical bonding provided by EDFs is much richer than that provided by a single numerical electronegativity difference, for instance, since the complete distribution of probabilities enables for a fine characterization of the electronic properties of the groups under consideration.^25^

It is also worth noting other QTC-based methodologies that have proven valuable in the characterization of chemical bonding and will be considered herein. One such method is based on the topology of the electron localization function (ELF), which offers an insightful depiction of the system through chemically meaningful Lewis structures, including atomic cores, lone pairs (LPs), and covalent bonds.^29^ Note that this approach has been applied to the study of chemical bonding in xenonium(II) ions containing the teflate group.^30^ Another significant tool is the noncovalent interactions (NCI) index, which enables the visualization of noncovalent interactions in real space.^31^ The NCI index has been effectively applied to a wide range of systems, from organic compounds to (organo)metallic complexes.^32^

As previously mentioned, in this contribution we utilize these methods to analyze the pentafluoroorthotellurate/fluoride analogy and to gain a deeper understanding of the intrinsic structure of the teflate group, which is eventually compared to other exceptionally electron-withdrawing groups. To the best of our knowledge, QCT methodologies (such as EDFs and IQA) have not yet been systematically applied to understanding the properties of teflate and their relationship to other electron-withdrawing functional groups. Such analysis holds significant potential for advancing our understanding of this ligand and for guiding the design of new teflate-containing systems.

## Results and Discussion

### Electronic Properties of the Pentafluoroorthotellurate Group

To undertake the investigation of the electronic structure of the pentafluoroorthotellurate (teflate) ligand we have selected a series of homologous teflate-based compounds that allows for an easy comparison. In this regard, the hypohalites XOTeF_5_ (X = F, Cl, Br, I) provide a simple, albeit chemically relevant, set in which the properties of the teflate group can be compared just as a function of the X group (Chart 1). All these compounds have been prepared in the bulk,^33−37^ yet IOTeF_5_ is unstable at room temperature and decomposes to I_2_ and I(OTeF_5_)_3_.^35^ Among this family of compounds, FOTeF_5_ and ClOTeF_5_ are probably the most interesting ones, since fluorinated hypofluorites and hypochlorites behave as strong oxidizers with diverse applications in organic synthesis.^38−43^ In fact, both have been used to prepare teflate-containing organic compounds.^44−46^ The hypochlorite species, ClOTeF_5_, which is an important teflate-transfer reagent of increasing interest nowadays, has been of most synthetic use within this set of species, as the partially positively charged Cl atom can react with species containing E–Cl bonds (E = element) to form the corresponding E–OTeF_5_ compounds upon release of Cl_2_.^7,8,47−54^

The geometrical structures of XOTeF_5_ (X = F, Cl, Br, I) systems is comparable in all cases (Figure S1), and render three different types of fluorine atoms within the teflate scaffold, which are referred to as F_a_, F_b_ and F_c_ (see Chart 1). The Te–F (185 pm) and Te–O distances (between 197 pm for X = F and 190 pm for X = I) are nearly independent of the X group. Note that, while the Te–F distance agrees well with that computed on the basis of covalent radii (187 pm), the Te–O distance is in all cases slightly longer than the expected value for a single bond (185 pm).^55^ As expected, the O–X distance is highly dependent on X, being 142, 169, 184, and 200 pm for X = F, Cl, Br and I, respectively. Such values are also significantly longer than those expected for single O–X bonds, which are 116, 152, 166, and 186 pm for X = F, Cl, Br and I, respectively.

The QTAIM charges reveal that the teflate moiety is considerably ionic, as unraveled by the very high positive charge of Te (between 3.63 and 3.67 |e^–^|, depending on the system) and the significantly negative charges of the fluorine atoms (F_a_–F_c_) bonded to it (between −0.65 and −0.62 |e^–^|), as shown in Table S2. These individual charges are comparable to previous reports on Hg,^56,57^ Xe,^30^ C and B-teflate compounds,^58^ although all these works considered NPA, not QTAIM charges. Nonetheless, NPA charges are generally comparable to QTAIM ones in polar systems. Namely, the Te charges are higher to 3.2 |e^–^| and lower than 4.0 |e^–^| in all cases, those of F being about −0.6 |e^–^|. These also agrees with the electrostatic potential surfaces (ESP) shown in Figure S2, which show negative regions in F atoms. It is worth noting that the total charge of TeF_5_ remains nearly independent of X, varying only slightly between 0.51 (X = F) and 0.46 |e^–^| (X = I), as shown in Table 1. On the contrary, the charge of the O atom is much more affected by the nature of X: while it shows negative values in all cases, the absolute value increases from X = F (−0.37 |e^–^|) to I (−1.01 |e^–^|), as the electronegativity of X decreases. In line with this result, the charge of X evolves from negative in the case of F (−0.14 |e^–^|) to positive in all the other cases – ranging from 0.29 |e^–^| in the case of Cl to 0.55 |e^–^| for I. Overall, the teflate group acquires a negative charge for X = Cl (−0.29 |e^–^|), Br (−0.40 |e^–^|) and I (−0.61 |e^–^|), while it is positive for X = F (+0.14 |e^–^|). These results points to the teflate group being more electronegative than Cl, Br and I, while slightly less than F. In broad terms, we justify the constant charge of TeF_5_ by considering that the Te atom is bonded to five terminal fluorine atoms, which are highly electronegative. This results in the Te atom bearing a very high positive charge (higher than 3 |e^–^|, which corresponds to less than one valence electron available in Te). Consequently, bonding with an additional electronegative atom, such as O, does not substantially alter its charge. This interpretation is in line with the very high ionic character of Te–F interaction, as unraveled by IQA, EDF and ELF analysis (*vide infra*).

**Table 1:** QTAIM Charges (in |e^–^|) for XOTeF_5_, XF and XCl (X = F, Cl, Br, I)

	XOTeF_5_				XF	XCl
X =	*q*(X)	*q*(O)	*q*(TeF_5_)	*q*(OTeF_5_)	*q*(X)	*q*(X)
F	–0.14	–0.37	0.51	0.14	0.00	–0.39
Cl	0.29	–0.78	0.49	–0.29	0.39	0.00
Br	0.40	–0.88	0.48	–0.40	0.47	0.14
I	0.55	–1.01	0.46	–0.55	0.58	0.31

For comparison, QTAIM charges are evaluated alongside those obtained for the XF and XCl series (X = F, Cl, Br, I). To facilitate the reader’s understanding, from now on we will refer to the above-mentioned set of systems as XY, with X = F, Cl, Br, I; and Y = OTeF_5_, F, Cl. For XF, the charges of X displace to more positive values (when compared with XOTeF_5_), the difference with XOTeF_5_ systems decreasing when decreasing the electronegativity of X. For example, for X = F, *q*(X) increases from −0.14 to 0.0 |e^–^|, while for X = I, it increases only marginally, from 0.55 to 0.58 |e^–^|. This suggests that fluoride and teflate exhibit greater electronic similarity when bonded to less electronegative ligands. For XCl, the opposite trend is observed, that is, *q*(X) shifts to less positive values than those in the teflate-derivatives, which agrees with chloride being less electronegative than both, fluoride and teflate. This result correlates with our previous studies on Mn and Co-based systems.^7,8^

**Figure 2:**
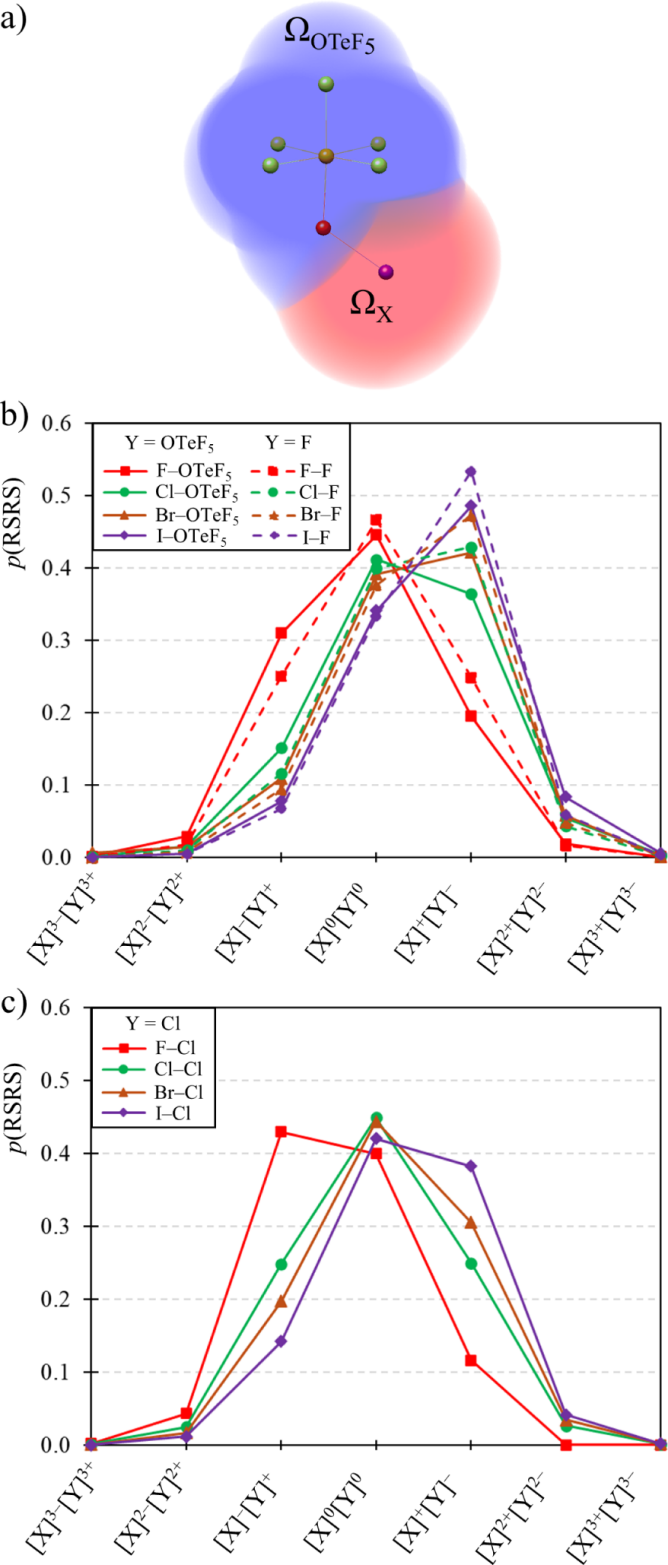
(a) 2-basin partition for XOTeF_5_ systems into Ω_X_ and  basins. 2-basins EDFs for XY systems (X = F, Cl, Br, I), (b) Y = OTeF_5_ (solid lines) and Y = F (dashed lines), and (c) Y = Cl.

To attain a more detailed picture on the electron distribution within these systems, we resorted to an EDF analysis.^24^ The systems were partitioned into two different QTAIM basins: X and Y groups, as shown in Figure 2a for XOTeF_5_. EDF results for XY systems when Y = OTeF_5_ (solid lines) and Y = F (dashed lines) are provided in Figure 2b. Several patterns are revealed. On the one side, for X = F (that is, for the FOTeF_5_ and F_2_ systems), the main RSRS is the neutral one, [F]^0^[Y]^0^. The probabilities of these RSRSs are 0.445 (Y = OTeF_5_) and 0.466 (Y = F). For FOTeF_5_, the following relevant RSRS is that in which the F atom acquires an additional electron, [F]^−^[OTeF_5_]^+^, with a probability of 0.310, followed by the opposite situation, [F]^+^[OTeF_5_]^−^, with a significantly lower probability of 0.195. The other alternatives, [F]^2–^[OTeF_5_]^2+^ and [F]^2+^[OTeF_5_]^2–^, show residual probabilities lower than 0.03. Overall, this probability distribution, with a peak at the neutral distribution and a significant shift to [F]^−^[OTeF_5_]^+^, is indicative of a polar covalent bond and shows that the F atom is more prone to attracting electrons to itself than OTeF_5_, that is, they are more electronegative. In this regard, the comparison with F_2_, a homopolar diatomic molecule with a purely covalent bonding regime and a perfectly symmetric EDF profile with respect to the neutral RSRS, is illustrative.

When we consider X = Cl, Br and I (and Y = OTeF_5_ and F), the EDF profile shifts to situations in which the Y group acquires additional electrons, favoring [X]^δ+^[Y]^δ−^ RSRSs. For X = Cl, the probability peak depends on Y; namely, for ClOTeF_5_, it corresponds to [Cl]^0^[OTeF_5_]^0^, with *p* = 0.412, closely followed by [Cl]^+^[OTeF_5_]^−^ (*p* = 0.364). Broadly speaking, ClF exhibits a mirror distribution, with *p* = 0.429 for [Cl]^+^[F]^−^ and *p* = 0.399 for [Cl]^0^[F]^0^. Consistent with the above-mentioned findings, this difference highlights the higher electronegative character of fluorine when compared to teflate. For both X = Br and I, the main RSRS involves the loss of one electron by X, [X]^+^[Y]^−^ (Y = OTeF_5_, F), followed by [X]^0^[Y]^0^.

We now compare the previous results with those obtained for Y = Cl (Figure 2c). At first sight, Cl has a much lower tendency to host electrons than OTeF_5_ and F, as revealed by EDF profiles shifted to RSRSs in which the Cl atom accommodates less electrons than F and OTeF_5_. This is especially clear in the BrY and IY systems. While for Y = OTeF_5_ and F the most probable RSRS are those in which Y acquires an additional electron, [X]^+^[Y]^−^, for Y = Cl, the main RSRS is [X]^0^[Y]^0^. This way, the EDF analysis suggests that the teflate group is significantly more electronegative than chloride, while less than fluoride, in agreement with the interpretation emerging from the QTAIM charges alone.

The energetic counterpart of the interactions is evaluated by resorting to IQA and delocalization indices (DIs). The latter represent the number of electron pairs shared by two basins and are considered as the real space analog of the bond order concept.^59^ Moreover, the DI is directly related to the covalent *V*_xc_ term of the IQA scheme, in such a way that, to a zeroth order approximation, , thus also being an energy descriptor.^60^ Conversely, the *V*_cl_ term is linked to electrostatic interactions and, in the same zeroth order approximation, it can be computed by resorting to Coulomb’s law: , where *q*_1_ and *q*_2_ are the atomic charges of the two groups that interact.^61^ It is to be stressed that in very polar, yet neutral systems, the *V*_cl_ terms are large and canceling. This translates into the total interaction energy, *E*_int_, being highly affected by the long-range character of electrostatic interactions. As a result, *V*_xc_ has been proposed to be a better measure of bond strength than *E*_int_.^18^ In this regard, we comment on *V*_cl_ and *V*_xc_ independently, but less attention is paid to *E*_int_.

The X–Y interaction energy terms in XY species (X = F, Cl, Br, I; Y = OTeF_5_, F, Cl) are provided in Table 2. Within each series (Y = OTeF_5_, F, Cl), *V*_xc_ decreases in absolute value (i.e., it is less favorable) when going from X = F to X = I. If we compare F–F and F–OTeF_5_, we can see that teflate and fluoride analogues lead to very similar values of *V*_xc_ (−224.4 and −215.4 kcal·mol^–1^, respectively). Moreover, they show small and positive values of *V*_cl_ (21.9 and 34.5 kcal·mol^–1^, respectively), in such a way that the overall interaction energy is negative (−202.6 and −180.9 kcal·mol^–1^, respectively) due to *V*_xc_ being higher in absolute value than *V*_cl_. The positive value of *V*_cl_ but overall attractive interaction energy is indicative of covalent bonding regimes, pointing toward both groups having comparable electron withdrawing properties, as reported for Co and Mn monomeric analogues.^7,8^

**Table 2:** *V*_xc_ and *V*_cl_ IQA Interaction Terms (Values in kcal·mol^–1^) and Delocalization Indices for X–Y Interactions in XY (X = F, Cl, Br, I; Y = OTeF_5_, F, Cl) Systems

	*V*_xc_			*V*_cl_			DI		
X =	Y = OTeF_5_	Y = F	Y = Cl	Y = OTeF_5_	Y = F	Y = Cl	Y = OTeF_5_	Y = F	Y = Cl
F	–224.4	–215.4	–189.8	21.9	34.5	–55.1	1.38	1.28	1.26
Cl	–209.6	–189.8	–188.2	–11.2	–55.1	27.0	1.47	1.26	1.43
Br	–178.9	–162.6	–169.0	–42.4	–85.8	14.9	1.37	1.17	1.39
I	–150.2	–138.8	–148.3	–84.6	–124.3	–17.1	1.25	1.08	1.32

Across the entire XY set of compounds, the *V*_xc_ term is consistently more negative for teflate-containing systems, indicating a stronger electron-sharing contribution in the bonding. This is also revealed by the delocalization indices (DIs), which are slightly higher for OTeF_5_ structures. As expected, the *V*_cl_ term is negative for X = Cl, Br, I, since X and Y fragments have opposite-sign charges. Noteworthy, it is significantly more attractive for XF analogues than for XOTeF_5_ and XCl, revealing a stronger electrostatic contribution to the bonding, because of the more pronounced tendency of fluoride to attract electrons, which leads to a higher charge separation.

The comparison of XCl with XOTeF_5_ reveals that the F–Cl interaction is much more electrostatic than F–OTeF_5_, as represented by a negative value of *V*_cl_ (−55.1 vs 21.9 kcal·mol^–1^, respectively), and a significantly less attractive *V*_xc_ term (−189.8 vs −224.4 kcal·mol^–1^, respectively). This points to less electron sharing between both groups, as also supported by a decrease in the DI from 1.38 (F–OTeF_5_) to 1.26 (F–Cl).

Overall, the results indicate that the teflate group exhibits a high electron-withdrawing character, comparable yet slightly smaller than that of fluorine, but significantly larger than that of chlorine.

### Electronic Structure of the Pentafluoroorthotellurate Group

We now delve into the structure of the OTeF_5_ group by analyzing the interaction energy terms between the several components that form it. First, we observe that the interactions within the TeF_5_ subgroup of teflate are largely unaffected by the identity of the X atom. In contrast, the interaction involving the O atom is significantly influenced by the nature of X, consistent with the QTAIM charge analysis results. This way, we start by describing TeF_5_ interactions and, given their robustness with respect to the system, we base our following discussion on FOTeF_5_. The detailed results for the whole set of compounds, including FOTeF_5_, are provided in the Supporting Information (Tables S3–S6).

With respect to Te–F_a_, Te–F_b_ and Te–F_c_ interactions, the *V*_cl_ and *V*_xc_ values for the three cases are nearly identical and are therefore explained collectively through average values, leaving the individual data to Table S3. Given the large positive charge of Te and the negative charge of fluorine atoms (F_a_, F_b_ and F_c_), it is not surprising that the *V*_cl_ term (∼−440 kcal·mol^–1^) be about four times higher in absolute value than the *V*_xc_ one (∼−107 kcal·mol^–1^), respectively pointing to a major electrostatic contribution to the Te–F bonding.

**Figure 3:**
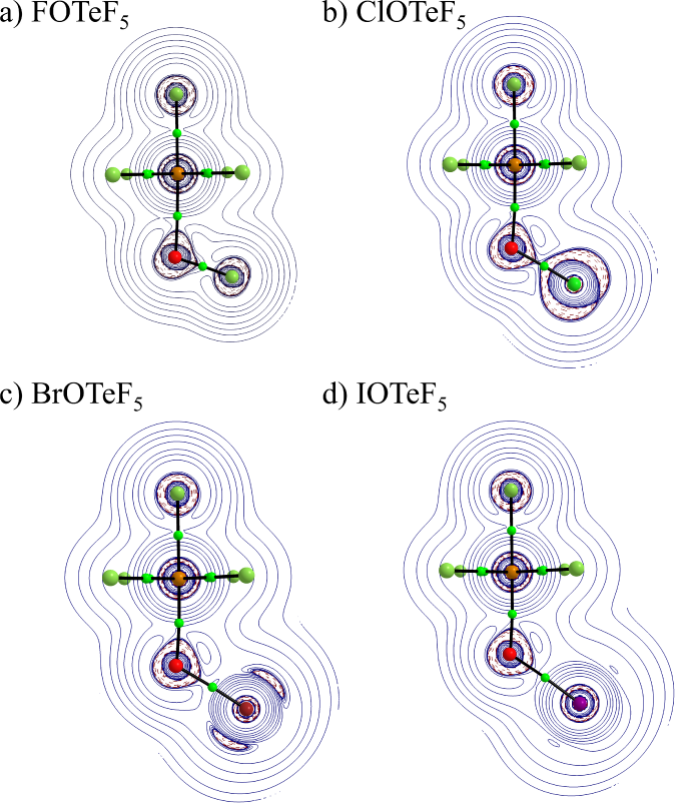
Laplacian of the electron density isocontours (isovalue = 0.001 au) for XOTeF_5_ (X = F, Cl, Br, I) systems on a plane containing X, O, Te and F_a_ atoms (solid-blue positive, dashed-red, negative). Bond critical points are shown in green.

**Figure 4:**
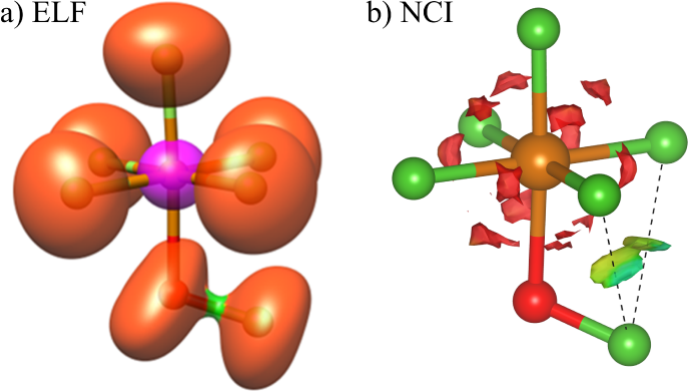
ELF (a) and NCI plot (b) for FOTeF_5_. The ELF isovalue is 0.60, core basin of Te are depicted in purple, monosynaptic basins in orange and disynaptic V(O,F) basin in green. NCI plot corresponds to a reduced density gradient of 0.65 au, and is colored in the [−0.03, 0.03] au range of sign(λ_2_)·ρ.

The previous result agrees with the analysis of the Laplacian of ρ (Figure 3), in which the lone pairs (LPs) of F_a_ atoms are clearly shown (see Figure S3 for lone pairs in F_b_ and F_c_) as nonbonded charge accumulation around atomic centers (depicted in red). The relatively high electron density at the Te–F_a_ bond critical point (BCP) of 0.159 au and the positive value of ∇^2^ρ (0.585 au) also support a close shell (ionic) interaction, which is extensive to interactions with F_b_ and F_c_ (Table S7). The ELF picture also points in this direction (Figure 4a), as shown by the absence of any disynaptic V(Te,F) basin – representing the covalent bonding between Te and F atoms – and an overall population of 7.8 *e* in monosynaptic V(F) basins – representing LPs of F atoms. Nonetheless, the covalent (electron sharing) contribution to the bonding should not be disregarded, as shown by non-negligible *V*_xc_ interaction energy terms, and relatively high DIs (0.63).

Given the large individual charges of the F atoms within the teflate structure (F_a_, F_b_ and F_c_), it is not surprising that 1,3 interactions between them be relevant. As for Te–F interactions, they are virtually independent of the X group, and thus we only comment in the text on the values for F–OTeF_5_ (Tables S3–S6). There are six potential F–F interactions within the teflate structure: F_a_–F_b_, F_a_–F_c_, F_b_–F_b_, F_c_–F_c_ and two types of F_b_–F_c_, depending on whether the F atoms are in *cis* (F_b_–F_c_) or in *trans* (F_b_–F_c_′) positions. In this regard, note that we use a hyphen (−) to represent interactions. However, this should not be interpreted as indicating a classical (covalent) F–F bond. Instead, it signifies the potential interaction between the two atoms under study. The same convention applies to other interactions, such as Te–X, as discussed below (*vide infra*). Since all F atoms bear negative charges, the *V*_*cl*_ term is repulsive, taking values of about 55 kcal·mol^–1^ for all interactions but for F_b_–F_c_′ (38.4 kcal·mol^–1^), the exact values being provided in Table S3. This last result is expected because the atoms involved are further apart compared to those of the other interactions, and classical Coulomb interactions decrease as the distance between charges increases.

The *V*_xc_ term is much smaller in absolute value, about −9 kcal·mol^–1^ for all cases but F_b_–F_c_′ (−0.4 kcal·mol^–1^). Nonetheless, it is not negligible, revealing electron sharing interactions between F atoms. Such result is also revealed by the DIs, which take a value of 0.10–0.11 in all cases. Be that as it may, given that the dominant term is *V*_cl_, the overall F–F interaction energy is repulsive: about 46 kcal·mol^–1^ for all cases but F_b_–F_c_′ (38.0 kcal·mol^–1^). This overall repulsive interaction between F atoms can also be visualized in real space by the NCI index, as shown by red isosurfaces between them (see Figure 4b for X = F systems, and Figure S5 for X = Cl, Br and I).

As introduced above, while interactions within the TeF_5_ subgroup are very robust with respect to the halogen atom (X) in the XOTeF_5_ family, those involving O and X atoms are significantly affected by the nature of X (see Table 3). With respect to the O–X interaction, the *V*_xc_ term is more favorable for X = F (−211.9 kcal·mol^–1^), decreasing in absolute value as we move down the halogen group, which is related with the higher covalent character of the O–F interaction with respect to heavier halides. In this direction, the classical interaction *V*_cl_ shows the opposite behavior: it is positive (28.7 kcal·mol^–1^) for X = F, and increasingly negative for X = Cl (−68.4 kcal·mol^–1^), Br (−111.2 kcal·mol^–1^) and I (−167.3 kcal·mol^–1^), as the atomic charge of such groups becomes more positive and the charge separation is higher. When examining the DIs, it may seem unusual that the DI for X = Cl is higher than that for X = F. This behavior can be explained by the fact that π orbitals of F atoms are energetically less available for bonding, meaning that the X–O bond is mainly σ in character. In contrast, for X = Cl, π orbitals are more available to interact with O, providing an additional contribution to the DI. This is revealed by the natural adaptive orbitals (NAdOs), which show higher occupation numbers for O–X π orbitals when X = Cl than when X = F, see Figure S6 and Table S8.^62^

**Table 3:** *V*_cl_ and *V*_xc_ IQA Interaction Terms (in kcal·mol^–1^) and Delocalization Indices for O–X, Te–O and Te–X Interactions in XOTeF_5_ (X = F, Cl, Br, I) Systems

	X =	*V*_*cl*_	*V*_*xc*_	DI
O–X	F	28.7	–211.9	1.24
	Cl	–68.4	–195.8	1.28
	Br	–111.2	–165.9	1.19
	I	–167.3	–138.0	1.07
Te–O	F	–277.5	–103.9	0.64
	Cl	–502.8	–109.4	0.66
	Br	–559.1	–113.4	0.68
	I	–632.3	–118.8	0.71
Te–X	F	–46.7	–3.7	0.04
	Cl	163.6	–3.6	0.05
	Br	209.6	–3.1	0.05
	I	268.6	–2.7	0.05

The inspection of the Laplacian of the electron density of the O–X bond (Figure 3) shows that the distortion from sphericity that justifies the presence of the oxygen’s two LPs decreases from X = F to X = I. This implies that the stereoactivity of these LPs should also decrease in the same order, with possible consequences in the structure of the solvation layers in solution. The distortion of the oxygen’s LPs is also revealed by the ELF (Figure 4a). This behavior is compatible with the increasing negative charge of the O atom, which smoothly approaches a more spherical entity with less condensed Lewis pairs.

With respect to the Te–O interaction, we can see that the *V*_*xc*_ dependency of X is very loose: it ranges between −103.9 kcal·mol^–1^ for X = F and −118.8 kcal·mol^–1^ for X = I. In this regard, the same behavior is found for the DIs, which range between 0.64 (X = F) and 0.71 (X = I). On the contrary, *V*_*cl*_ is highly dependent on X, because of the increasing charge difference between O and X atoms when going down the halogen group (Table 1). Namely, it shifts from −277.5 kcal·mol^–1^ for X = F to −632.3 kcal·mol^–1^ for X = I. This supports that the bonding has a roughly fixed covalent component and an X-dependent electrostatic contribution. The latter depends on the charge of the O atom, which in turn depends on the nature of X. This way, less electronegative groups lead to a more negative charge on O, and thus a more attractive electrostatic interaction.

Given the significant charge separation within teflate-containing systems, the 1,3 and 1,4 interactions involving X and O atoms are significant, similarly to the F–F interactions observed in the TeF_5_ substructure. IQA and DI values for Te–X interactions are presented in Table 3. The interaction is predominantly electrostatic, as evidenced by the negligible magnitude of *V*_xc_ compared to *V*_cl_ in all cases. In this regard, it is not unexpected that *V*_cl_ be negative (stabilizing, *aka* attractive) for X = F and increasingly positive (destabilizing, *aka* repulsive) for X = Cl, Br and I, respectively, as the latter show increasingly positive charges. The electron-sharing *V*_xc_ interaction is very small, ranging from −3.7 kcal·mol^–1^ (X = F) to −2.7 kcal·mol^–1^ (X = I), in agreement with DIs of 0.04–0.05.

Other relevant 1,3 long-range interactions involve O–F_a/b/c_ and X–F_a/b/c_ (see Tables S3–S6 for IQA and DI values). Both are dominated by *V*_cl_, consistent with a main electrostatic component. In this regard, O–F_a/b/c_ are repulsive, in line with the negative charge of O and F atoms. Remarkably the *V*_xc_ component is barely independent of the nature of X, contrarily to *V*_cl_. For instance, for FOTeF_5_ the *V*_xc_ for the O–F_a_ interaction takes a negligible value of −0.4 kcal·mol^–1^, (DI = 0.01), while the O–F_b_ and O–F_c_ interactions take much higher values: −7.4 and −9.4 kcal·mol^–1^, respectively.

In broad strokes, the same behavior is observed for 1,4 X–F_a/b/c_ interactions. Nonetheless, the different sign of *q*(X) for X = F (negative) and X = Cl, Br and I (positive) translates into *V*_*cl*_ being slightly positive for X = F and negative for the other cases. Moreover, the *V*_xc_ term shows a different nature for X–F_a_ and X–F_c_ interactions, for which it is negligible (lower than −1.0 kcal·mol^–1^), and for X–F_b_. The latter exhibits a non-negligible value of about −4.0 kcal·mol^–1^ (DI about 0.05). The latter interactions can also be unraveled by the NCI method (see Figure 4b for the case of X = F, and Figure S5 for the whole set of systems). They appear as two disk-shaped isosurfaces colored in green, which is indicative of weak localized interactions, in agreement with the IQA analysis.^32^

**Figure 5:**
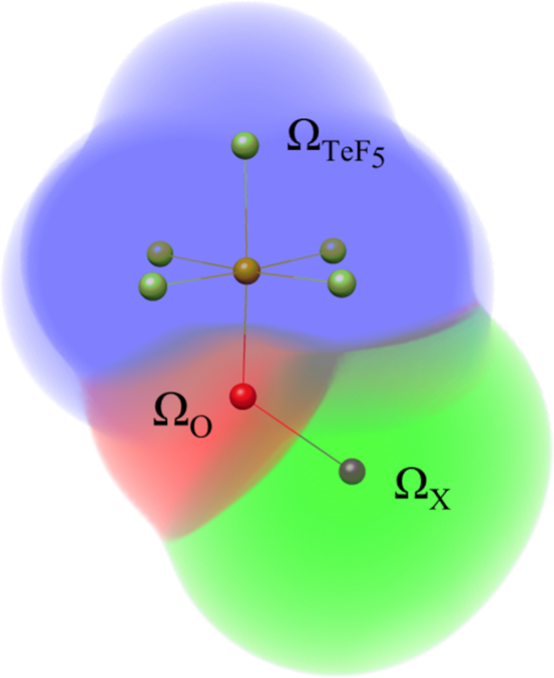
Real space representation of the three QTAIM basins (TeF_5_, O and X) considered for performing three-basin EDFs analysis.

Finally, to attain a more detailed picture of the electron distribution between X, O and TeF_5_ fragments, we resorted to a three-basin EDF analysis considering the real space partition within these three subgroups, as shown in Figure 5. The main results are provided in Table 4 (see Tables S10–S13 for the whole set of probabilities). At first sight, the TeF_5_ subgroup acts as a sink of electrons in favor of O and X atoms. In this regard, the most probable RSRS for X = F (*p* = 0.229), Cl (*p* = 0.211) and Br (*p* = 0.198) is that in which TeF_5_ donates one electron to the O atom, i.e., [X]^0^[O]^−^[TeF_5_]^+^; while this is the third RSRS in relevance for X = I (*p* = 0.172). The second RSRS in probability terms for X = F is [X]^−^[O]^0^[TeF_5_]^+^ (*p* = 0.165), which sharply decreases in relevance for X = Cl (*p* = 0.08), Br (*p* = 0.062) and I (*p* = 0.041), given the much lower electronegativity of the latter. It is also relevant to note that the most populated RSRS for X = I (*p* = 0.231), [X]^+^[O]^2–^[TeF_5_]^+^, is the fifth in relevance for X = F (*p* = 0.094), while it is the second for X = Cl (*p* = 0.175) and Br (*p* = 0.198).

**Table 4:** Probability of Selected RSRS (the Six More Relevant in FOTeF_5_) in the Three-Basin EDFs Analysis of XOTeF_5_ Systems (X = F, Cl, Br, I)^a^

	X =			
RSRS	F	Cl	Br	I
[X]^0^[O]^−^ [TeF_5_]^+^	0.229 [1]	0.211 [1]	0.198 [1]	0.172 [3]
[X]^−^ [O]^0^[TeF_5_]^+^	0.165 [2]	0.080 [5]	0.062 [5]	0.041 [6]
[X]^0^[O]^0^[TeF_5_]^0^	0.152 [3]	0.138 [3]	0.130 [4]	0.113 [4]
[X]^−^[O]^+^[TeF_5_]^0^	0.100 [4]	0.046 [6]	0.036 [7]	0.023 [12]
[X]^+^[O]^2–^[TeF_5_]^+^	0.094 [5]	0.175 [2]	0.198 [2]	0.231 [1]
[X]^+^[O]^−^ [TeF_5_]^0^	0.070 [6]	0.130 [4]	0.148 [3]	0.172 [2]

a The relevance order of each RSRS is indicated in square brackets.

Overall, these results further support that the TeF_5_ moiety of teflate tends to lose electrons with respect to the neutral fragments, while the O atom tends to accumulate them.

### Comparison with Related Highly Electron-Withdrawing O-Donor Systems

**Chart 2:**
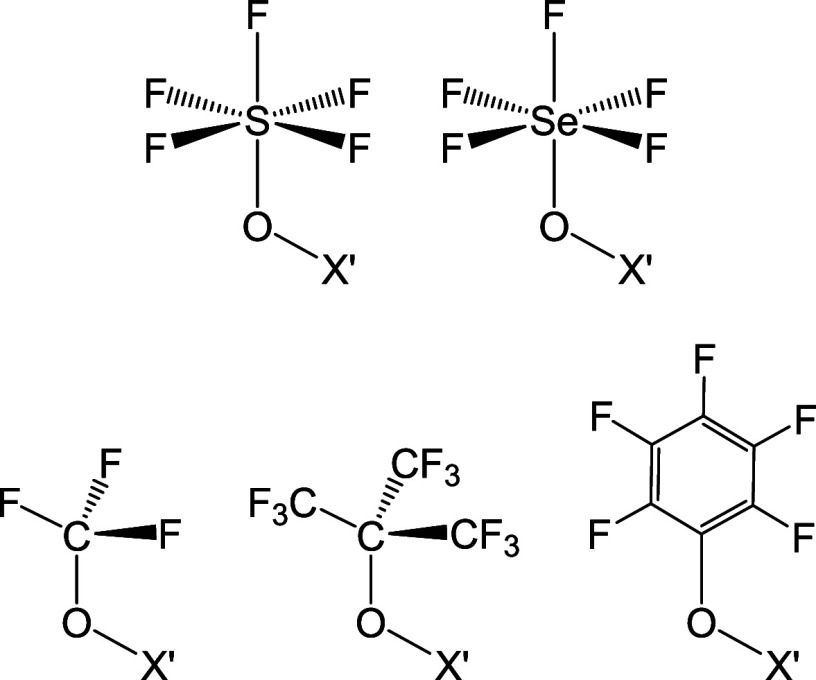
Selected X′Y′ systems for comparison with teflate analogues. X′ = OTeF_5_, F, Cl, Br, I; Y′ = OTeF_5_, OSF_5_, OSeF_5_, OCF_3_, OC(CF_3_)_3_, OC_6_F_5_ (structures indicated)

Having obtained a comprehensive understanding of the structure of the teflate group and the factors influencing its unique electronic properties, in this section we compare it with other related relevant systems. The selected groups are shown in Chart 2, and include fluorinated O-donor systems such as −OSF_5_, −OSeF_5_, −OCF_3_, −OC(CF_3_)_3_ and −OC_6_F_5_, which have been reported to exhibit also very high electron-withdrawing ability and electronic properties similar to those of the teflate group.^1,3,63,64^ The following notation is considered: X′Y′, with X′ = OTeF_5_, F, Cl, Br, I and Y′ = OTeF_5_, OSF_5_, OSeF_5_, OCF_3_, OC(CF_3_)_3_, OC_6_F_5_.

The QTAIM charges for OTeF_5_–Y′ systems are provided in Table 5 (first column). The charges of the Y′ group are 0.0 |e^–^| for Y″ = OSF_5_, and −0.01 |e^–^| for Y″ = OSeF_5_, indicating that these groups, which are the most similar to teflate since we are just varying the central Group-16 element, exhibit nearly identical group electronegativities. The high group electronegativity of the three OEF_5_ moieties (E = S, Se, Te) has already been pointed out in previous reports,^1,5^ having been claimed that OSeF_5_ is slightly more electron withdrawing than OTeF_5_.^64^ The charges for Y′ = OCF_3_ and OC(CF_3_)_3_ are slightly positive (0.02 |e^–^|), suggesting that these groups are less electronegative than teflate. For Y″ = OC_6_F_5_, the charge is significantly positive (0.10 |e^–^|), indicating that its difference in electronegativity with teflate is more pronounced. The same conclusions can be drawn by examining the halogen series: X” = F, Cl, Br, I (see Table 5 second and third columns for X′ = F and Cl, respectively, and Table S16 for X′ = Br and I). Namely, the charges of Y′ are positive for X′ = F, and negative for X′ = Cl, Br, I, the absolute values following the aforementioned order. Moreover, this shows that the electronegativity of all the groups lie between that of fluoride and chloride.

**Table 5:** QTAIM Charges (in |e^–^|) for Selected X′Y′ Systems

	*q*(Y′)		
Y′ =	X′ = OTeF_5_	X′ = F	X′ = Cl
OTeF_5_	0.00	0.14	–0.29
OSF_5_	0.00	0.14	–0.29
OSeF_5_	–0.01	0.13	–0.30
OCF_3_	0.02	0.15	–0.27
OC(CF_3_)_3_	0.02	0.15	–0.26
OC_6_F_5_	0.10	0.22	–0.17

A more detailed understanding of the electron distribution within the X′Y′ series was obtained through EDF analysis. Specifically, we employed a 2-basin QTAIM partitioning, dividing the system into X′ and Y′ groups, similar to Figure 2a. The distributions of the most probable RSRSs for X′ = OTeF_5_ and F are provided in Table 6. Regarding the OTeF_5_–Y′ series, the [OTeF_5_]^0^[Y′]^0^ RSRS is consistently the most probable configuration, showing nearly identical probabilities for Y′ = OTeF_5_ (*p* = 0.429), OSF_5_ (*p* = 0.429), and OSeF_5_ (*p* = 0.426). For Y′ = OSF_5_, the second most relevant RSRS is [OTeF_5_]^−^[Y′]^+^ (*p* = 0.249), followed closely by [OTeF_5_]^+^[Y′]^−^ (*p* = 0.247). In contrast, for Y′ = OSeF_5_, the order reverses, with [OTeF_5_]^+^[Y′]^−^ (*p* = 0.249) slightly more probable than [OTeF_5_]^−^[Y′]^+^ (*p* = 0.245). Nonetheless, the differences are minimal. This suggests a nearly identical statistical distribution of electrons within the groups, reflecting similar group electronegativities for the three OEF_5_ fragments (E = S, Se, Te).

There is a significant difference when the previous systems are compared with Y′ = OCF_3_, OC(CF_3_)_3_ and OC_6_F_5_, since the probability distribution shifts toward [OTeF_5_]^0^[Y′]^0^ and [OTeF_5_]^−^[Y′]^+^ RSRSs, at the expense of [OTeF_5_]^+^[Y′]^−^. For example, the probability of the latter RSRS (ranked second in the three cases), is 0.244 for Y′ = OCF_3_, 0.243 for Y′ = OC(CF_3_)_3_ and 0.218 for OC_6_F_5_. As tentatively proposed in terms of the QTAIM charges, the EDFs predict that OCF_3_ and OC(CF_3_)_3_ groups are slightly less electronegative than OTeF_5_, and this difference is more pronounced in the case of OC_6_F_5_.

**Table 6:** Probability of Selected RSRS (the Three Most Relevant) in the Two-Basin EDFs Analysis of X′Y′OTeF_5_ Systems for X′ = OTeF_5_ and F

		Y’ =					
	RSRS	OTeF_5_	OSF_5_	OSeF_5_	OCF_3_	OC(CF_3_)_3_	OC_6_F_5_
OTeF_5_–Y′	[OTeF_5_]^0^[Y′]^0^	0.429	0.429	0.426	0.439	0.431	0.432
	[OTeF_5_]^−^[Y′]^+^	0.248	0.249	0.245	0.252	0.253	0.278
	[OTeF_5_]^+^[Y′]^−^	0.248	0.247	0.249	0.244	0.243	0.218
F–Y′	[F]^0^[Y′]^0^	0.445	0.446	0.445	0.449	0.443	0.443
	[F]^−^[Y′]^+^	0.310	0.309	0.307	0.314	0.315	0.349
	[F]^+^[Y′]^−^	0.195	0.196	0.197	0.192	0.191	0.166

The same general conclusions can be drawn when the comparison is performed in terms of the halogen derivatives, that is, for X′ = F, Cl, Br and I (see Table 6 for X= F and Tables S17–S21 for the whole set of compounds). While we do not comment the results in detail, it can be seen that, in general terms, the F-derivatives have an enhanced tendency to host electrons in the F atom. As a result, the [F]^−^[Y′]^+^ RSRS is favored over [F]^+^[Y′]^−^. Moreover, the EDF distribution is nearly identical for Y′ = OTeF_5_, OSF_5_ and OSeF_5_; while OCF_3_ and OC(CF_3_)_3_ comparatively favor [F]^−^[Y′]^+^ RSRSs, and such resonance structure is pronouncedly more favored for OC_6_F_5_.

Given the electronic similarity between OTeF_5_, OSeF_5_ and OSF_5_ groups, we expect that they lead to comparable interaction energies when bonded to the same functional group. In this context, we calculated X′–Y′ IQA interaction energy and DIs for the whole X’Y’ series (Tables S22–S26) Briefly, both terms (*V*_xc_ and *V*_cl_) for the X′–Y′ interaction for Y’ = OTeF_5_, OSF_5_, OSeF_5_ are very similar in all cases (X′ = OTeF_5_, F, Cl, Br, I). The fact that the three teflate-related groups lead to similar values of *V*_*xc*_ indicates that they have similar group electronegativities, which translates into similar electron-sharing properties. In this regard, we note that the X′–Y’ distances (Figure S14) are very similar for Y′ = OTeF_5_, OSF_5_, and OSeF_5_; and they progressively increase for Y′ = OCF_3_, OC(CF_3_)_3_, and OC_6_F_5_, with the increase being most pronounced in the latter case. This trend aligns with the previous discussion. The *V*_cl_ term takes positive (repulsive) values for all the OTeF_5_–Y′ and F–Y′ interactions, but the overall interaction energy is negative (attractive). This is a clear distinctive feature of covalent bonding and indicates that the electronic properties of the six groups under consideration are similar enough to form a covalent bond with the extraordinary electronegative OTeF_5_ and F.

## Conclusions

Using state-of-the-art computational methods, the exceptionally high electron-withdrawing ability of the pentafluoroorthotellurate group is rationalized. In fact, its group electronegativity is similar to that of the related OSeF_5_ and OSF_5_ groups, and higher than that of OCF_3_, OC(CF_3_)_3_ and OC_6_F_5_. Importantly, the electron-withdrawing tendency of teflate is comparable to that of fluorine, albeit a bit less pronounced. This way, the teflate ligand might allow for the stabilization of rare species, as has already been demonstrated by many examples throughout the periodic table.^1,2^ In this regard, it holds a privileged position within the family of fluorinated O-donor ligands, the explanation for which is 3-fold: (a) its group electronegativity is higher than that of other common ligands including O–C bonds; (b) the OTeF_5_ moiety is much more easily accessible than the lighter yet similarly electron-withdrawing analogues OSeF_5_ and OSF_5_, which has led to a comparatively more significant progress of teflate chemistry; (c) teflate compounds are usually the most stable within the OEF_5_ series (E = S, Se, Te). The results arising from our investigation demonstrate once more that teflate is the best analogue of fluoride in terms of electronic properties in comparison to other highly electronegative O-donor ligands. Therefore, teflate compounds are especially suited for future research in different areas of fluorine chemistry, which range from the synthesis of Lewis superacids or very strong oxidizers, to spectroscopic investigations shedding light into electronic properties of 3d metal complexes, yet inaccessible because of the polymeric nature of metal fluorides.

The TeF_5_ moiety in the teflate group is very robust with respect to the group bonded to it and in this regard, the O atom plays a key role in modulating the charge, and, in general, the properties of the OTeF_5_ group as a whole. This implies that the choice of the teflate-transfer reagent used to obtain a target teflate compound is of particular relevance. For example, within the herein investigated hypohalite series, ClOTeF_5_ is the reagent of choice due to the partial positive charge of the Cl atom and indeed its use has increased especially in the last years. Therefore, our investigation can help not only understand why the teflate gives such special properties to its compounds, but also which might be the most suitable reactions, in each particular case, for the formation of a particular element–OTeF_5_ bond.

Our results also underscore the value of the QCT methodologies used in this study, providing a thorough, nuanced, and intuitive understanding of chemical systems while maintaining physical rigor. In fact, it is shown that this combination of QCT methods can help experimentalists understand the properties of their systems and therefore design molecules with target properties and even envision a suitable synthetic route to prepare them.

## Computational Details

Geometry optimizations and wave function calculations were performed at the Density Functional Theory (DFT) level, by means of the GGA hybrid B3LYP exchange-correlation functional;^65^ in conjunction with D3BJ empirical correction dispersion scheme,^66^ and def2-TZVP basis set;^67^ as implemented in the Gaussian 16 software package.^68^

QTAIM and IQA calculations were carried out with AIMALL.^69^ The ELF was obtained with TopMod package,^70^ with a grid of 150 × 150 × 150 points, representations being performed by using UCSF Chimera.^71^ The electron distribution functions were computed with the EDF program.^28^ NCI plots were obtained by means of NCIPLOT4,^72^ graphical representations being performed with VESTA.^73^

## Supporting Information Available

Geometries of XY and X′Y′ sets of systems, atomic charges, IQA interaction energies, NCI plots, NAdOs, electron density and its Laplacian, ELF and ESP representations (PDF)

## References

[ref1] Seppelt K. Stabilization of Unusual Oxidation and Coordination States by the Ligands OSF_5_, OSeF_5_, and OTeF_5_. Angew. Chem., Int. Ed. Engl. 1982, 21, 877–888. 10.1002/anie.198208773.

[ref2] Gerken M.; Mercier H. P. A.; Schrobilgen G. J. Advanced Inorganic Fluorides: Synthesis, Characterization and Applications, Nakajima T.; Zemva B.; Tressaud A., Eds.; Elsevier: Lausanne, 2000, pp. 117–174.

[ref3] Riddlestone I. M.; Kraft A.; Schaefer J.; Krossing I. Taming the Cationic Beast: Novel Developments in the Synthesis and Application of Weakly Coordinating Anions. Angew. Chem., Int. Ed. Engl. 2018, 57, 13982–14024. 10.1002/anie.201710782. 29266644

[ref4] Seppelt K.; Lentz D. Extremely High Electronegativities. Angew. Chem., Int. Ed. 1978, 17, 355–356. 10.1002/anie.197803551.

[ref5] Lentz D.; Seppelt K. Pentafluorotellurate(VI) des fünfwertigen Iod; die Elektronegativität der Gruppen OTeF_5_ und OSeF_5_. Z. Anorg. Allg. Chem. 1980, 460, 5–16. 10.1002/zaac.19804600101.

[ref6] Birchall T.; Myers R. D.; DeWaard H.; Schrobilgen G. J. Multinuclear NMR and Moessbauer study of pentafluorooxotellurate (OTeF_5_) derivatives of tellurium, iodine, and xenon: Spectroscopic determination of the relative electronegativities of fluoride and pentafluorooxotellurate. Inorg. Chem. 1982, 21, 1068–1073. 10.1021/ic00133a039.

[ref7] Pérez-Bitrián A.; Munárriz J.; Sturm J. S.; Wegener D.; Krause K. B.; Wiesner A.; Limberg C.; Riedel S. Further Perspectives on the Teflate versus Fluoride Analogy: The Case of a Co(II) Pentafluoroorthotellurate Complex. Inorg. Chem. 2023, 62, 12947–12953. 10.1021/acs.inorgchem.3c01730. 37505485

[ref8] Pérez-Bitrián A.; Munárriz J.; Krause K. B.; Schlögl J.; Hoffmann K. F.; Sturm J. S.; Hadi A. N.; Teutloff C.; Wiesner A.; Limberg C.; Riedel S. Questing for homoleptic mononuclear manganese complexes with monodentate O-donor ligands. Chem. Sci. 2024, 15, 5564–5572. 10.1039/D4SC00543K. 38638238 PMC11023055

[ref9] Miller P. K.; Abney K. D.; Rappé A. K.; Anderson O. P.; Strauss S. H. Electronic and molecular structure of pentafluorooxotellurate(1). Inorg. Chem. 1988, 27, 2255–2261. 10.1021/ic00286a010.

[ref10] Popelier P. L. A. Intermolecular Forces and Clusters I; Springer: Berlin, Heidelberg, 2005.

[ref11] Popelier P. L. A. Applications of topological methods in molecular chemistry; Springer International Publishing: Berlin, Heidelberg, 2016.

[ref12] Matta C. F. How dependent are molecular and atomic properties on the electronic structure method? Comparison of Hartree-Fock, DFT, and MP2 on a biologically relevant set of molecules. J. Comput. Chem. 2010, 31, 1297–1311. 10.1002/jcc.21417. 19882732

[ref13] Martín Pendás A.; Francisco E.; Suárez D.; Costales A.; Díaz N.; Munárriz J.; Rocha-Rinza T.; Guevara-Vela J. M. Atoms in molecules in real space: A fertile field for chemical bonding. Phys. Chem. Chem. Phys. 2023, 25, 10231–10262. 10.1039/D2CP05540F. 36994471

[ref14] Bader R. F. Atoms in Molecules: A Quantum Theory; Oxford University press: Oxford, 1990.

[ref15] aPopelier P. L. A. The QTAIM Perspective of Chemical Bonding. In The Chemical Bond: Fundamental Aspects of Chemical Bonding, 1st ed.; John Wiley & Sons, 2014; pp. 271–308.

[ref16] Landeros-Rivera B.; Gallegos M.; Munárriz J.; Laplaza R.; Contreras-García J. New venues in electron density análisis. Phys. Chem. Chem. Phys. 2022, 24, 21538–21548. 10.1039/D2CP01517J. 36069366

[ref17] Blanco M. A.; Martín Pendás A.; Francisco E. Interacting Quantum Atoms: A Correlated Energy Decomposition Scheme Based on the Quantum Theory of Atoms in Molecules. J. Chem. Theory Comput. 2005, 1, 1096–1109. 10.1021/ct0501093. 26631653

[ref18] Pendás M. A.; Casals-Sainz J. L.; Francisco E. On Electrostatics, Covalency, and Chemical Dashes: Physical Interactions versus Chemical Bonds. Chem. -Eur. J. 2019, 25, 309–314. 10.1002/chem.201804160. 30264915

[ref19] aGuevara-Vela J. M.; Romero-Montalvo E.; Costales A.; Martín Pendás Á.; Rocha-Rinza T. The nature of resonance-assisted hydrogen bonds: A quantum chemical topology perspective. Phys. Chem. Chem. Phys. 2016, 18, 26383–26390. 10.1039/C6CP04386K. 27435637

[ref20] aNiyas M. A.; Ramakrishnan R.; Vijay V.; Sebastian E.; Hariharan M. Anomalous Halogen–Halogen Interaction Assists Radial Chromophoric Assembly. J. Am. Chem. Soc. 2019, 141, 4536–4540. 10.1021/jacs.8b13754. 30740979

[ref21] aGuevara-Vela J. M.; Hess K.; Rocha-Rinza T.; Martín Pendás A.; Flores-Álamo M.; Moreno-Alcántar G. Stronger-together: The cooperativity of aurophilic interactions. Chem. Commun. 2022, 58, 1398–1401. 10.1039/D1CC05241A. 34994363

[ref22] aMunarriz J.; Velez E.; Casado M. A.; Polo V. Understanding the reaction mechanism of the oxidative addition of ammonia by (PXP)Ir(i) complexes: The role of the X group. Phys. Chem. Chem. Phys. 2018, 20, 1105–1113. 10.1039/C7CP07453K. 29238771

[ref23] Guevara-Vela J. M.; Francisco E.; Rocha-Rinza T.; Martín Pendás Á. Interacting Quantum Atoms—A Review. Molecules 2020, 25, 4028 10.3390/molecules25174028. 32899346 PMC7504790

[ref24] aMartín Pendás A.; Francisco E.; Blanco M. A. Spin resolved electron number distribution functions: How spins couple in real space. J. Chem. Phys. 2007, 127, 144103 10.1063/1.2784392. 17935382

[ref25] Pendás M. Á.; Francisco E. Chemical Bonding from the Statistics of the Electron Distribution. ChemPhysChem 2019, 20, 2722–2741. 10.1002/cphc.201900641. 31270916

[ref26] Casals-Sainz J. L.; Jara-Cortés J.; Hernández-Trujillo J.; Guevara-Vela J. M.; Francisco E.; Martín Pendás Á. Exotic Bonding Regimes Uncovered in Excited States. Chem. -Eur. J. 2019, 25, 12169–12179. 10.1002/chem.201902369. 31310392

[ref27] Barrena-Espés D.; Munárriz J.; Martín Pendás Á. How electrons still guard the space: Electron number distribution functions based on QTAIM∩ELF intersections. J. Chem. Phys. 2024, 160, 144106 10.1063/5.0199318. 38591678

[ref28] aFrancisco E.; Martín Pendás A.; Blanco M. A. Electron number probability distributions for correlated wave functions. J. Chem. Phys. 2007, 126, 094102 10.1063/1.2709883. 17362099

[ref29] aSavin A.; Nesper R.; Wengert S.; Fassle T. F. ELF: The Electron Localization Function. Angew. Chem., Int. Ed. 1997, 36, 1808–1832. 10.1002/anie.199718081.

[ref30] Mercier H. P. A.; Moran M. D.; Sanders J. C. P.; Schrobilgen G. J.; Suontamo R. J. Synthesis, Structural Characterization, and Computational Study of the Strong Oxidant Salt [XeOTeF_5_][Sb(OTeF_5_)_6_]·SO_2_ClF. Inorg. Chem. 2005, 44, 49–60. 10.1021/ic0400890. 15627360

[ref31] Johnson E. R.; Keinan S.; Mori-Sánchez P.; Contreras-García J.; Cohen A. J.; Yang W. Revealing Noncovalent Interactions. J. Am. Chem. Soc. 2010, 132, 6498–6506. 10.1021/ja100936w. 20394428 PMC2864795

[ref32] Laplaza R.; Peccati F.; A. Boto R.; Quan C.; Carbone A.; Piquemal J. −. P.; Maday Y.; Contreras-García J. NCIPLOT and the analysis of noncovalent interactions using the reduced density gradient. Wiley Interdiscip. Rev.: Comput. Mol. Sci. 2020, 11, e1497 10.1002/wcms.1497.

[ref33] Schack C. J.; Wilson W. W.; Christe K. O. Synthesis and Characterization of TeF_5_OF. Inorg. Chem. 1983, 22, 18–21. 10.1021/ic00143a005.

[ref34] Schack C. J.; Christe K. O. An improved synthesis of tellurium fluoride hypofluorite (TeF_5_OF). Inorg. Chem. 1984, 23, 2922 10.1021/ic00187a004.

[ref35] Seppelt K.; Nothe D. Stability of xenon(II) compounds. Pentafluorooxyselenium and pentafluorooxytellurium radicals. Bis(pentafluorotellurium) peroxide and chlorine pentafluoroorthotellurate. Inorg. Chem. 1973, 12, 2727–2730. 10.1021/ic50129a046.

[ref36] Schack C. J.; Christe K. O. New syntheses of pentafluorotellurium hypochlorite. J. Fluorine Chem. 1982, 21, 393–396. 10.1016/S0022-1139(00)81525-0.

[ref37] Seppelt K. Neue Derivate der Pentafluoroorthoselensäure, Halogenderivate der Pentafluoroorthotellursäure. Chem. Ber 1973, 106, 1920–1926. 10.1002/cber.19731060621.

[ref38] Shreeve J. M. Fluorinated Hypofluorites and Hypochlorites. Adv. Inorg. Chem. Radiochem. 1983, 26, 119–168. 10.1016/S0898-8838(08)60092-6.

[ref39] Hesse R. H. Application of Fluoroxy Compounds to Organic Synthesis: Electrophilic Fluorination of Unsaturated Molecules. Isr. J. Chem. 1978, 17, 60–70. 10.1002/ijch.197800009.

[ref40] Kollonitsch J. Novel Methods for Selective Fluorination of Organic Compounds: Design and Synthesis of Fluorinated Antimetabolites. Isr. J. Chem. 1978, 17, 53–59. 10.1002/ijch.197800008.

[ref41] Schack C. J.; Christe K. O. Reactions of Electropositive Chlorine Compounds with Fluorocarbons. Isr. J. Chem. 1978, 17, 20–30. 10.1002/ijch.197800004.

[ref42] Barton D. H. R. The invention of reactions useful for the synthesis of specifically fluorinated natural products. Pure Appl. Chem. 1977, 49, 1241–1249. 10.1351/pac197749091241.

[ref43] Barton D. H. R. New methods of specific fluorination. Pure Appl. Chem. 1970, 21, 285–293. 10.1351/pac197021020285. 5458820

[ref44] Schack C. J.; Christe K. O. Reactions of TeF_5_OCl with fluorocarbon iodides and synthesis of CF_3_OTeF_5_. J. Fluorine Chem. 1990, 47, 79–87. 10.1016/S0022-1139(00)80449-2.

[ref45] Schack C. J.; Christe K. O. Reactions of pentafluorotellurium hypohalites with fluoroolefins. J. Fluorine Chem. 1984, 24, 467–476. 10.1016/S0022-1139(00)83167-X.

[ref46] Schack C. J.; Christe K. O. Pentafluorotelluriumoxide derivatives of fluorocarbons. J. Fluorine Chem. 1988, 39, 153–162. 10.1016/S0022-1139(00)82773-6.

[ref47] Fischer L.; Hoffmann K. F.; Riedel S. A Rare Example of a Gallium-based Lewis Superacid: Synthesis and Reactivity of Ga(OTeF_5_)_3_. Chem. -Eur. J. 2024, 30, e202403266 10.1002/chem.202403266. 39298145

[ref48] Drews T.; Seppelt K. Fe(OTeF_5_)_3_, Darstellung, Struktur und Reaktivität. Z. Anorg. Allg. Chem. 1991, 606, 201–207. 10.1002/zaac.19916060120.

[ref49] Lentz D.; Seppelt K. OTeF_5_-Verbindungen von P, As und Sb. Z. Anorg. Allg. Chem. 1983, 502, 83–88. 10.1002/zaac.19835020712.

[ref50] Turowsky L.; Seppelt K. Rheniumverbindungen mit dem Liganden -OTeF_5_. Z. Anorg. Allg. Chem. 1990, 590, 37–47. 10.1002/zaac.19905900105.

[ref51] Turowsky L.; Seppelt K. Molybdän- und Wolframverbindungen mit dem Liganden -OTeF_5_. Z. Anorg. Allg. Chem. 1990, 590, 23–36. 10.1002/zaac.19905900104.

[ref52] Vij A.; Wilson W. W.; Vij V.; Corley R. C.; Tham F. S.; Gerken M.; Haiges R.; Schneider S.; Schroer T.; Wagner R. I. Methyl Tin(IV) Derivatives of HOTeF_5_ and HN(SO_2_CF_3_)_2_: A Solution Multinuclear NMR Study and the X-ray Crystal Structures of (CH_3_)_2_SnCl(OTeF_5_) and [(CH_3_)_3_Sn(H_2_O)_2_][N(SO_2_CF_3_)_2_]. Inorg. Chem. 2004, 43, 3189–3199. 10.1021/ic049785q. 15132626

[ref53] Pérez-Bitrián A.; Hoffmann K. F.; Krause K. B.; Thiele G.; Limberg C.; Riedel S. Unravelling the Role of the Pentafluoroorthotellurate Group as a Ligand in Nickel Chemistry. Chem. -Eur. J. 2022, 28, e202202016 10.1002/chem.202202016. 35851723 PMC9825845

[ref54] Winter M.; Peshkur N.; Ellwanger M. A.; Pérez-Bitrián A.; Voßnacker P.; Steinhauer S.; Riedel S. Gold Teflates Revisited: From the Lewis Superacid [Au(OTeF_5_)_3_] to the Anion [Au(OTeF_5_)_4_]^−^. Chem. -Eur. J. 2023, 29, e202203634 10.1002/chem.202203634. 36598847

[ref55] Pyykkö P. Additive Covalent Radii for Single-, Double-, and Triple-Bonded Molecules and Tetrahedrally Bonded Crystals: A Summary. J. Phys. Chem. A 2015, 119, 2326–2337. 10.1021/jp5065819. 25162610

[ref56] De Backere J. R.; Mercier H. P. A.; Schrobilgen G. J. Thiazyl Trifluoride (NSF_3_) Adducts and Imidodifluorosulfate (F_2_OSN^–^) Derivatives of Hg(OTeF_5_)_2_. Inorg. Chem. 2015, 54, 9989–10000. 10.1021/acs.inorgchem.5b01769. 26413855

[ref57] De Backere J. R.; Mercier H. P. A.; Schrobilgen G. J. Noble-Gas Difluoride Complexes of Mercury(II): The Syntheses and Structures of Hg(OTeF_5_)_2_·1.5NgF_2_ (Ng = Xe, Kr) and Hg(OTeF_5_)_2_. J. Am. Chem. Soc. 2014, 136, 3888–3903. 10.1021/ja412193z. 24490719

[ref58] Moran M. D.; Mercier H. P. A.; Schrobilgen G. J. Synthesis and Structural Characterization of C(OTeF_5_)_4_ and a Comparative Structural Study of the Isoelectronic B(OTeF_5_)_4_^–^ Anion. Inorg. Chem. 2007, 46, 5034–5045. 10.1021/ic700362g. 17503812

[ref59] Fradera X.; Austen M. A.; Bader R. F. W. The Lewis Model and Beyond. J. Phys. Chem. A 1999, 103, 304–314. 10.1021/jp983362q.

[ref60] Francisco E.; Menéndez-Crespo D.; Costales A.; Martín Pendás Á. A multipolar approach to the interatomic covalent interaction energy. J. Comput. Chem. 2017, 38, 816–829. 10.1002/jcc.24758. 28211059

[ref61] Martín Pendás A.; Francisco E. Real space bond orders are energetic descriptors. Phys. Chem. Chem. Phys. 2018, 20, 16231–16237. 10.1039/C8CP02485E. 29863214

[ref62] aFrancisco E.; Martín Pendás A.; García-Revilla M.; Álvarez Boto R. A Hierarchy of Chemical Bonding Indices in Real Space from Reduced Density Matrices and Cumulants. Comput. Theor. Chem. 2013, 1003, 71–78. 10.1016/j.comptc.2012.09.009.

[ref63] Elinburg J. K.; Doerrer L. H. Synthesis, structure, and electronic properties of late first-row transition metal complexes of fluorinated alkoxides and aryloxides. Polyhedron 2020, 190, 114765 10.1016/j.poly.2020.114765.

[ref64] Seppelt K. Recent Developments in the Chemistry of Some Electronegative Elements. Acc. Chem. Res. 1979, 12, 211–216. 10.1021/ar50138a004.

[ref65] Becke A. D. A new mixing of Hartree–Fock and local density-functional theories. J. Chem. Phys. 1993, 98, 1372–1377. 10.1063/1.464304.

[ref66] aGrimme S.; Antony J.; Ehrlich S.; Krieg H. A consistent and accurate ab initio parametrization of density functional dispersion correction (DFT-D) for the 94 elements H-Pu. J. Chem. Phys. 2010, 132, 154104 10.1063/1.3382344. 20423165

[ref67] Weigend F.; Ahlrichs R. Balanced basis sets of split valence, triple zeta valence and quadruple zeta valence quality for H to Rn: Design and assessment of accuracy. Phys. Chem. Chem. Phys. 2005, 7, 3297–3305. 10.1039/b508541a. 16240044

[ref68] Frisch M. J.; Trucks G. W.; Schlegel H. B.; Scuseria G. E.; Robb M. A.; Cheeseman J. R.; Scalmani G.; Barone V.; Petersson G. A.; Nakatsuji H., Gaussian 16, revision C.01; Gaussian, Inc.: Wallingford, CT, 2016.

[ref69] Todd A.; Keith T. K. AIMAll (Version 19.10.12). Gristmill Software: Overland Park KS, USA, 2019.

[ref70] Noury F. F. S.; Krokidis X.; Silvi B. The Topmod package. Laboratoire de Chime Theorique, 1997.

[ref71] aPettersen E. F.; Goddard T. D.; Huang C. C.; Couch G. S.; Greenblatt D. M.; Meng E. C.; Ferrin T. E. UCSF Chimera—A visualization system for exploratory research and analysis. J. Comput. Chem. 2004, 25, 1605–1612. 10.1002/jcc.20084. 15264254

[ref72] Boto R. A.; Peccati F.; Laplaza R.; Quan C.; Carbone A.; Piquemal J.-P.; Maday Y.; Contreras-García J. NCIPLOT4: Fast, Robust, and Quantitative Analysis of Noncovalent Interactions. J. Chem. Theory Comput. 2020, 16, 4150–4158. 10.1021/acs.jctc.0c00063. 32470306

[ref73] Momma K.; Izumi F. VESTA 3 for three-dimensional visualization of crystal, volumetric and morphology data. J. Appl. Crystallogr. 2011, 44, 1272–12. 10.1107/S0021889811038970.

