# Supplementary material for: Pentafluoroorthotellurate Uncovered: Theoretical Perspectives on an Extremely Electronegative Group
Source: Inorg Chem. 2025 Jan 3;64(2):1064–74. doi: 10.1021/acs.inorgchem.4c04603 (PMC11752526; doi:10.1021/acs.inorgchem.4c04603)
Supplement: Supplementary file 1 — ic4c04603_si_001.pdf [file ic4c04603_si_001.pdf]

Supporting Information for

Pentafluoroorthotellurate Uncovered: Theoretical Perspectives on an Extremely Electronegative Group

Daniel Barrena-Espés,[a] Ángel Martín Pendás,[a] Sebastian Riedel,[b] Alberto Pérez-Bitrián,[c,*] Julen Munárriz[d,*]

[a] Departamento de Química Física y Analítica, Universidad de Oviedo, Oviedo 33006, Spain.

[b] Fachbereich Biologie, Chemie, Pharmazie, Institut für Chemie und Biochemie – Anorganische Chemie, Freie Universität Berlin, Fabeckstraße 34/36, Berlin 14195, Germany.

[c] Institut für Chemie, Humboldt-Universität zu Berlin, Berlin 12489, Germany. Email: alberto.perez-bitrian@hu-berlin.de.

[d] Departamento de Química Física and Instituto de Biocomputación y Física de Sistemas Complejos (BIFI), Universidad de Zaragoza, Zaragoza 50009, Spain. E-mail: julen@unizar.es.

Geometries of XY systems

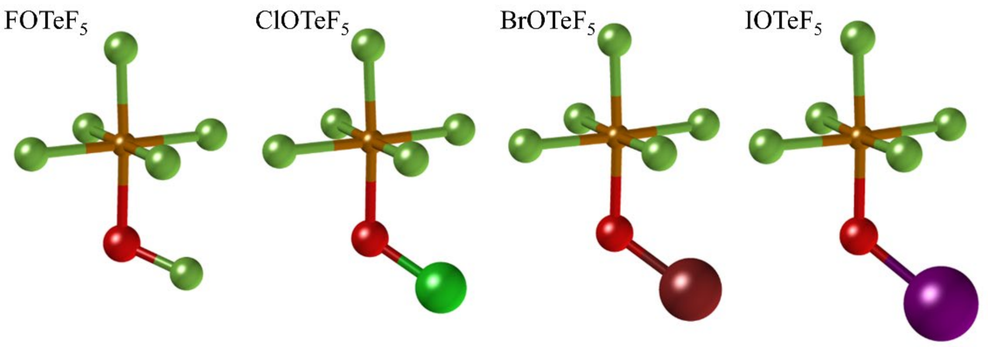

Figure S1. Geometries for $XOTeF_5$ (X = F, Cl, Br, I) systems optimized at the B3LYP-D3BJ/def2-SVP level of theory.

Table S1. Cartesian coordinates for XY (X = F, Cl, Br, I; Y = $OTeF_5$, F, Cl) systems optimized at the B3LYP-D3BJ/def2-SVP level of theory.

$FOTeF_5$

Te	0.205539	-0.002568	-0.000002
F	1.861766	-0.811372	0.000019
F	0.787932	1.184516	1.298543
F	-0.364388	-1.180706	1.310178
F	-0.364087	-1.180208	-1.310763
F	0.788202	1.185056	-1.297938
O	-1.519500	0.951062	-0.000046
F	-2.546317	-0.027839	0.000015

$ClOTeF_5$

Te	-0.415937	-0.001589	-0.000001
F	-2.085130	0.788753	-0.000226
F	-0.999978	-1.182940	1.304021
F	0.123613	1.198342	1.311501
F	0.123651	1.197691	-1.312084
F	-0.999809	-1.183618	-1.303486
O	1.289756	-0.927579	0.000280
Cl	2.697033	0.008190	0.000016

$BrOTeF_5$

Te	-0.796536	-0.003886	0.000003
F	-2.457561	0.806652	-0.000106
F	-1.400430	-1.174478	1.305298
F	-0.253285	1.196238	1.312655
F	-0.253475	1.195657	-1.313258
F	-1.400505	-1.175105	-1.304693
O	0.885910	-0.938577	0.000078
Br	2.463426	0.002000	0.000004

$IOTeF_5$

Te	-1.137132	-0.007851	0.000000
F	-2.795886	0.812297	0.000117
F	-1.756328	-1.168443	1.308259
F	-0.597703	1.198819	1.313031
F	-0.598006	1.198742	-1.313227
F	-1.756553	-1.168576	-1.308036
O	0.531418	-0.923203	-0.000152
I	2.309808	-0.001164	-0.000001

F_2

F	0.000000	0.000000	0.698295
F	0.000000	0.000000	-0.698295

ClF

Cl	0.000000	0.000000	0.568005
F	0.000000	0.000000	-1.072898

BrF

Br	0.000000	0.000000	0.362978
F	0.000000	0.000000	-1.411580

IF

I	0.000000	0.000000	0.279344
F	0.000000	0.000000	-1.645025

Cl_2

Cl	0.000000	0.000000	1.006770
Cl	0.000000	0.000000	-1.006770

BrCl

Cl	0.000000	0.000000	-1.457404
Br	0.000000	0.000000	0.707882

ICl

I	0.000000	0.000000	0.569401
Cl	0.000000	0.000000	-1.77519

QTAIM charges of $XOTeF_5$ systems

Table S2. QTAIM charges (in |e⁻|) for $XOTeF_5$ (X = F, Cl, Br, I) systems.

X =	q(X)	q(O)	q(Te)	q(F_a)	q(F_b)	q(F_c)	q(TeF_5)	q($OTeF_5$)
F	–0.14	–0.37	3.63	–0.62	–0.62	–0.63	0.51	0.14
Cl	0.29	–0.78	3.64	–0.63	–0.63	–0.63	0.49	–0.29
Br	0.40	–0.88	3.64	–0.63	–0.64	–0.63	0.48	–0.40
I	0.55	–1.01	3.65	–0.63	–0.65	–0.63	0.46	–0.55

ESP surfaces for $XOTeF_5$ systems

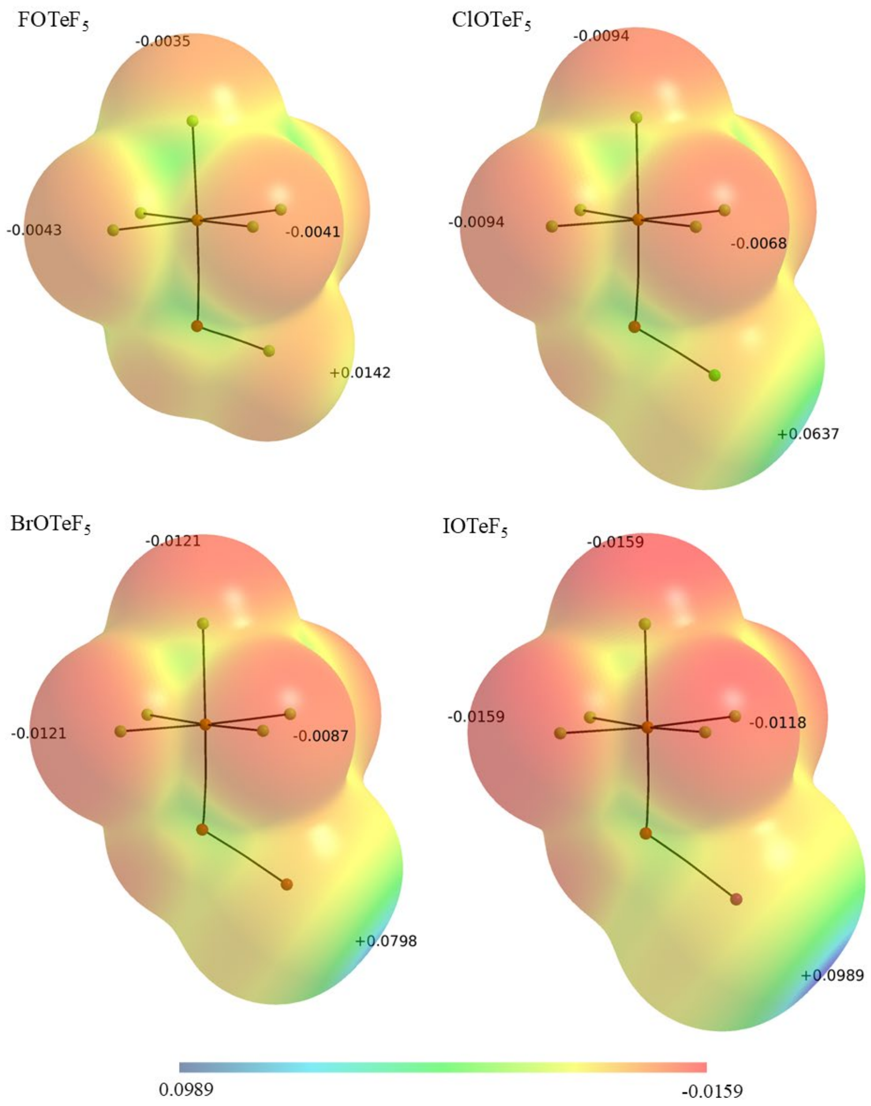

Figure S2. Molecular electrostatic potential (in au) for $XOTeF_5$ (X = F, Cl, Br, I) systems (electronic density isosurface of 0.001 au).

IQA interaction energies in $XOTeF_5$ systems

Table S3. IQA interaction terms (values in kcal·mol^{-1}) and delocalization indices for $X–OTeF_5$ when X = F.

Interaction X = F	E_{int}	V_{cl}	V_{xc}	DI
O–X	–183.1	28.7	–211.9	1.24
Te–O	–381.4	–277.5	–103.9	0.64
Te–X	–50.4	–46.7	–3.7	0.04
X–F_a	6.1	6.1	–0.1	0.00
X–F_b	7.0	10.7	–3.7	0.04
X–F_c	5.5	6.2	–0.7	0.02
O–F_a	23.7	24.1	–0.4	0.01
O–F_b	24.9	32.2	–7.4	0.10
O–F_c	27.5	36.8	–9.3	0.11
Te–F_a	–550.5	–442.8	–107.6	0.63
Te–F_b	–542.6	–435.5	–107.1	0.63
Te–F_c	–543.0	–436.8	–106.2	0.63
F_a–F_b	45.9	55.3	–9.4	0.10
F_a–F_c	46.0	55.4	–9.4	0.10
F_b–F_b	45.8	54.9	–9.1	0.10
F_c–F_c	45.9	55.8	–9.9	0.11
F_b–F_c	46.2	55.0	–8.8	0.10
F_b–$F_{c'}$	38.0	38.4	–0.4	0.10

Table S4. IQA interaction terms (values in kcal·mol^{-1}) and delocalization indices for X–$OTeF_5$ when X = Cl.

Interaction X = Cl	E_{int}	V_{cl}	V_{xc}	DI
O–X	–264.2	–68.4	–195.8	1.28
Te–O	–612.2	–502.8	–109.4	0.66
Te–X	160.0	163.6	–3.6	0.05
X–F_a	–16.0	–15.9	–0.1	0.00
X–F_b	–28.2	–24.0	–4.2	0.05
X–F_c	–22.1	21.3	–0.8	0.02
O–F_a	46.1	46.5	–0.4	0.01
O–F_b	58.3	66.3	–8.0	0.10
O–F_c	57.2	66.6	–9.4	0.10
Te–F_a	–550.0	–443.7	–106.3	0.63
Te–F_b	–545.2	–439.9	–105.3	0.62
Te–F_c	–544.1	–438.5	–105.6	0.62
F_a–F_b	46.8	56.4	–9.6	0.11
F_a–F_c	46.4	56.0	–9.6	0.11
F_b–F_b	47.5	56.6	–9.0	0.10
F_c–F_c	46.3	55.8	–9.5	0.11
F_b–F_c	47.1	55.9	–8.7	0.10
F_b–$F_{c'}$	38.7	39.0	–0.4	0.10

Table S5. IQA interaction terms (values in kcal·mol^{-1}) and delocalization indices for X–OTeF_5 when X = Br.

Interaction X = Br	E_{int}	V_{cl}	V_{xc}	DI
O–X	–277.0	–111.2	–165.9	1.19
Te–O	–672.5	–559.1	–113.4	0.68
Te–X	206.4	209.6	–3.1	0.05
X–F_a	–21.1	–21.1	–0.1	0.00
X–F_b	–36.7	–32.5	–4.2	0.05
X–F_c	–28.2	–27.4	–0.8	0.02
O–F_a	51.7	52.1	–0.4	0.01
O–F_b	66.6	75.0	–8.5	0.11
O–F_c	64.3	73.9	–9.5	0.10
Te–F_a	–550.2	–444.3	–105.9	0.62
Te–F_b	–547.2	–442.7	–104.5	0.62
Te–F_c	–545.3	–440.0	–105.2	0.62
F_a–F_b	47.2	56.9	–9.7	0.11
F_a–F_c	46.6	56.3	–9.7	0.11
F_b–F_b	48.4	57.3	–8.9	0.10
F_c–F_c	46.6	56.1	–9.5	0.10
F_b–F_c	47.7	56.3	–8.7	0.10
F_b–$F_{c'}$	39.0	39.4	–0.4	0.10

Table S6. IQA interaction terms (values in kcal·mol^{-1}) and delocalization indices for X–OTeF$_5$ when X = I.

Interaction X = I	E_{int}	V_{cl}	V_{xc}	DI
O–X	–305.3	–167.3	–138.0	1.07
Te–O	–751.1	–632.3	–118.8	0.71
Te–X	265.9	268.6	–2.7	0.05
X–F$_a$	–27.9	–27.8	–0.1	0.00
X–F$_b$	–47.7	–43.6	–4.0	0.05
X–F$_c$	–36.1	–35.4	–0.7	0.02
O–F$_a$	58.9	59.3	–0.5	0.01
O–F$_b$	77.4	86.5	–9.1	0.11
O–F$_c$	73.8	83.4	–9.6	0.11
Te–F$_a$	–550.0	–444.8	–105.2	0.62
Te–F$_b$	–549.7	–446.3	–103.3	0.61
Te–F$_c$	–546.9	–442.0	–104.9	0.62
F$_a$–F$_b$	47.8	57.6	–9.8	0.11
F$_a$–F$_c$	46.9	56.7	–9.9	0.11
F$_b$–F$_b$	49.6	58.5	–8.9	0.10
F$_c$–F$_c$	47.1	56.4	–9.3	0.10
F$_b$–F$_c$	48.4	57.0	–8.6	0.10
F$_b$–F$_{c'}$	39.5	39.9	–0.4	0.10

Laplacian of the electron density for XOTeF$_5$ systems

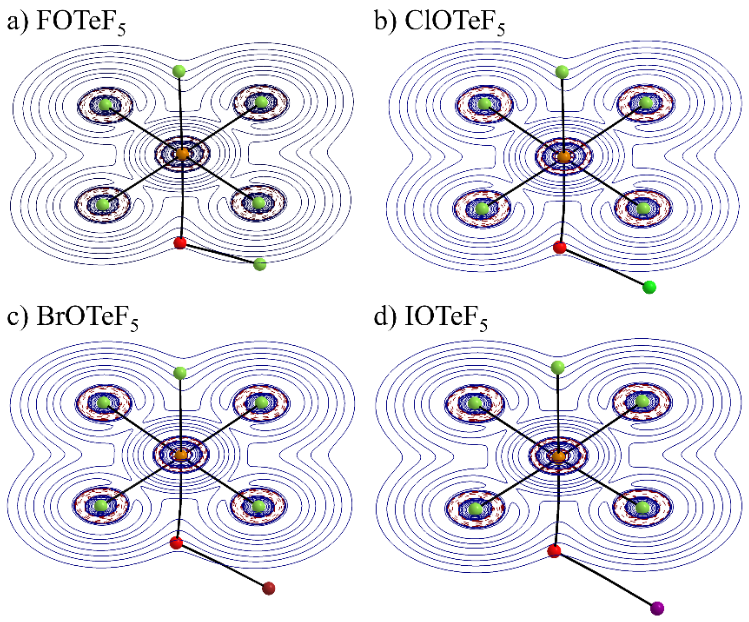

Figure S3. Alternative views of the Laplacian of the electron density for XOTeF$_5$ systems (X = F, Cl, Br, I) in which the lone pairs of F$_b$ and F$_c$ atoms are shown (isovalue = 0.001 au).

Table S7. ρ and $\nabla^2\rho$ values (in au) at bond critical points for $XOTeF_5$ systems (X = F, Cl, Br, I).

X =	F		Cl		Br		I	
BCP	ρ	$\nabla^2\rho$	ρ	$\nabla^2\rho$	ρ	$\nabla^2\rho$	ρ	$\nabla^2\rho$
Te–F_a	0.159	0.585	0.158	0.575	0.158	0.571	0.157	0.565
Te–F_b	0.156	0.560	0.156	0.558	0.155	0.558	0.155	0.557
Te–F_c	0.156	0.564	0.154	0.551	0.153	0.547	0.152	0.541
Te–O	0.140	0.246	0.146	0.298	0.150	0.327	0.155	0.369
O–X	0.284	0.305	0.206	–0.090	0.158	0.064	0.122	0.229

ELF for $XOTeF_5$ systems

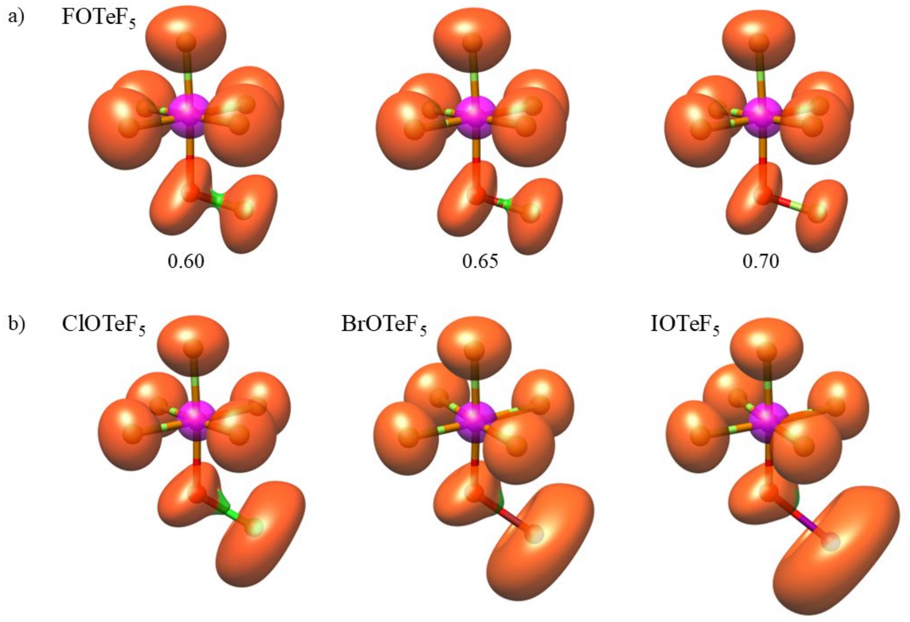

Figure S4. a) ELF for $FOTeF_5$ system with isovalues 0.60, 0.65 and 0.70. b) ELF representation for $XOTeF_5$ systems (X = Cl, Br, I) with isovalue 0.70.

NCI plot for $XOTeF_5$ systems

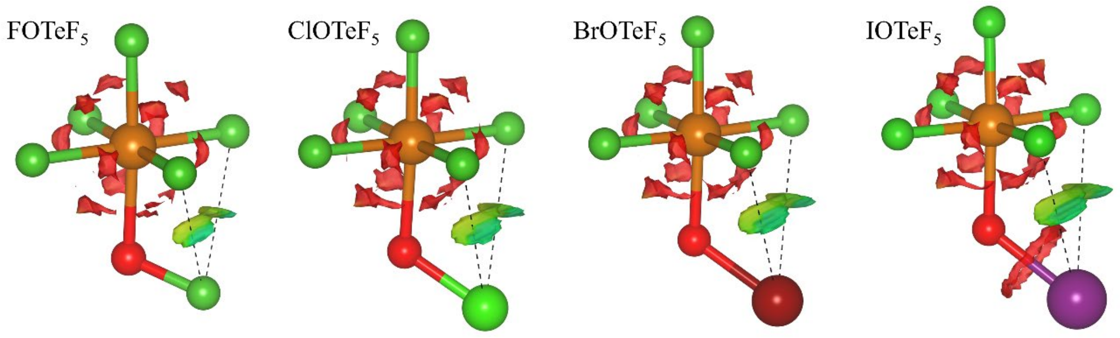

Figure S5. NCI plot for $XOTeF_5$ systems (X = F, Cl, Br, I). Surfaces are colored in the [−0.03, 0.03] au range of sign(λ_2)ρ (isosurface s = 0.65 au).

NAdOs for $XOTeF_5$ systems

O–X bond

Table S8. Eigenvalues associated with the most relevant two-fragment NAdOs for the O–X bond in the $XOTeF_5$ (X = F, Cl, Br, I) systems.

	X =			
Orbital	F	Cl	Br	I
σ	0.9548	0.9008	0.8370	0.7221
π_1	0.1124	0.1491	0.1373	0.1313
π_2	0.0696	0.0997	0.0866	0.0832
π^*	0.0577	0.0795	0.0760	0.0746

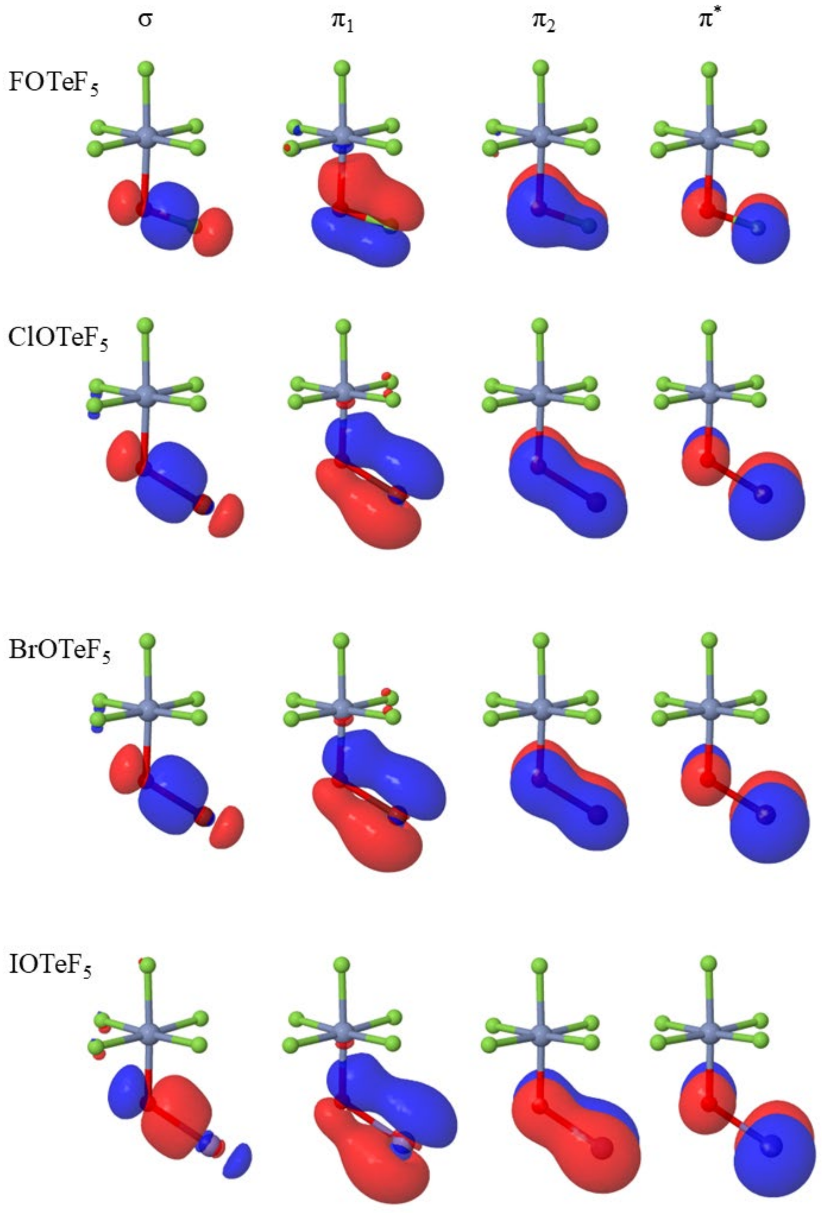

Figure S6. Representation of the most relevant two-fragment NAdOs involving the O–X bond (σ, π_1, π_2, π^*) for the $XOTeF_5$ (X = F, Cl, Br, I) systems (isovalue 0.70).

O–Te bond

Table S9. Eigenvalues associated with the most relevant two-fragment NAdOs for the O–Te bond in the $XOTeF_5$ (X = F, Cl, Br, I) systems.

	X =			
Orbital	F	Cl	Br	I
σ	0.588902	0.585073	0.591642	0.596880
π	0.109962	0.115675	0.121430	0.128529

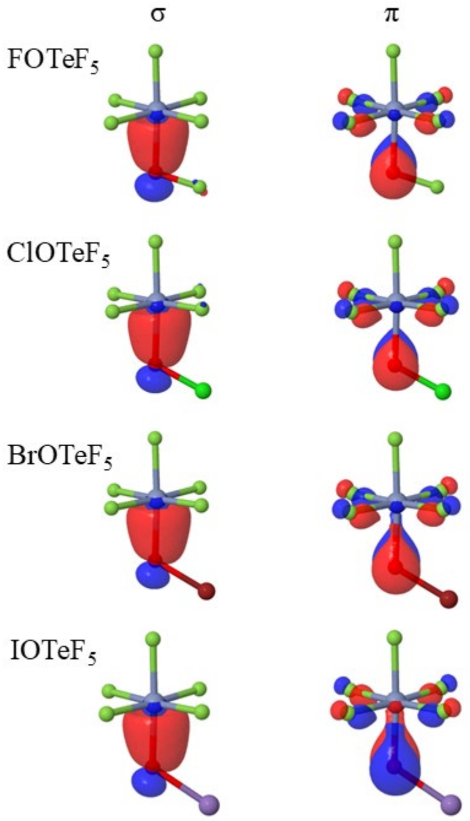

Figure S7. Representation of the most relevant two-fragment NAdOs involving the O–Te bond (σ and π) for the $XOTeF_5$ (X = F, Cl, Br, I) systems (isovalue 0.70).

EDFs for $XOTeF_5$ systems

Table S10. EDF probabilities for three-basin EDFs analysis of $XOTeF_5$ when X = F (probability threshold of 0.010).

RSRS	p(RSRS)
$[F]^0[O]^-[TeF_5]^+$	0.229
$[F]^-[O]^0[TeF_5]^+$	0.165
$[F]^0[O]^0[TeF_5]^0$	0.152
$[F]^-[O]^+[TeF_5]^0$	0.100
$[F]^+[O]^{2-}[TeF_5]^+$	0.094
$[F]^+[O]^-[TeF_5]^0$	0.070
$[F]^0[O]^+[TeF_5]^-$	0.034
$[F]^0[O]^{2-}[TeF_5]^{2+}$	0.026
$[F]^-[O]^-[TeF_5]^{2+}$	0.023
$[F]^-[O]^{2+}[TeF_5]^-$	0.019
$[F]^+[O]^0[TeF_5]^-$	0.019
$[F]^{2-}[O]^+[TeF_5]^+$	0.015

Table S11. EDF probabilities for three-basin EDFs analysis of $XOTeF_5$ when X = Cl (probability threshold of 0.010).

RSRS	p(RSRS)
$[Cl]^0[O]^-[TeF_5]^+$	0.211
$[Cl]^+[O]^{2-}[TeF_5]^+$	0.175
$[Cl]^0[O]^0[TeF_5]^0$	0.138
$[Cl]^+[O]^-[TeF_5]^0$	0.130
$[Cl]^-[O]^0[TeF_5]^+$	0.080
$[Cl]^-[O]^+[TeF_5]^0$	0.046
$[Cl]^+[O]^0[TeF_5]^-$	0.034
$[Cl]^0[O]^+[TeF_5]^-$	0.031
$[Cl]^0[O]^{2-}[TeF_5]^{2+}$	0.028
$[Cl]^{2+}[O]^{2-}[TeF_5]^0$	0.022
$[Cl]^+[O]^{3-}[TeF_5]^{2+}$	0.019
$[Cl]^{2+}[O]^{3-}[TeF_5]^+$	0.018
$[Cl]^-[O]^-[TeF_5]^{2+}$	0.014

Table S12. EDF probabilities for three-basin EDFs analysis of $XOTeF_5$ when X = Br (probability threshold of 0.010).

RSRS	*p*(RSRS)
$[Br]^0[O]^-[TeF_5]^+$	0.198
$[Br]^+[O]^{2-}[TeF_5]^+$	0.198
$[Br]^+[O]^-[TeF_5]^0$	0.148
$[Br]^0[O]^0[TeF_5]^0$	0.130
$[Br]^-[O]^0[TeF_5]^+$	0.062
$[Br]^+[O]^0[TeF_5]^-$	0.039
$[Br]^-[O]^+[TeF_5]^0$	0.036
$[Br]^0[O]^+[TeF_5]^-$	0.030
$[Br]^0[O]^{2-}[TeF_5]^{2+}$	0.028
$[Br]^{2+}[O]^{2-}[TeF_5]^0$	0.026
$[Br]^+[O]^{3-}[TeF_5]^{2+}$	0.023
$[Br]^{2+}[O]^{3-}[TeF_5]^+$	0.021
$[Br]^-[O]^-[TeF_5]^{2+}$	0.012
$[Br]^{2+}[O]^-[TeF_5]^-$	0.011

Table S13. EDF probabilities for three-basin EDFs analysis of $XOTeF_5$ when X = I (probability threshold of 0.010).

RSRS	*p*(RSRS)
$[I]^+[O]^{2-}[TeF_5]^+$	0.231
$[I]^+[O]^-[TeF_5]^0$	0.172
$[I]^0[O]^-[TeF_5]^+$	0.172
$[I]^0[O]^0[TeF_5]^0$	0.113
$[I]^+[O]^0[TeF_5]^-$	0.047
$[I]^-[O]^0[TeF_5]^+$	0.041
$[I]^{2+}[O]^{2-}[TeF_5]^0$	0.034
$[I]^{2+}[O]^{3-}[TeF_5]^+$	0.028
$[I]^+[O]^{3-}[TeF_5]^{2+}$	0.028
$[I]^0[O]^{2-}[TeF_5]^{2+}$	0.026
$[I]^0[O]^+[TeF_5]^-$	0.026
$[I]^-[O]^+[TeF_5]^0$	0.023
$[I]^{2+}[O]^-[TeF_5]^-$	0.015

Geometries of X'Y' systems

Table S14. Calculated X'–Y' distance (in Å) for X'Y' systems. Note that for X' = $OTeF_5$, F, Cl, Br and I the reported distance corresponds to O–O, O–F, O–Cl, O–Br and O–I, respectively.

Y' =	X' = $OTeF_5$	X' = F	X' = Cl	X' = Br	X' = I
$OTeF_5$	1.4196	1.4187	1.6900	1.8366	2.0032
OSF_5	1.4120	1.4129	1.6914	1.8393	2.0064
$OSeF_5$	1.4073	1.4110	1.6859	1.8345	2.0038
OCF_3	1.4297	1.4221	1.7013	1.8456	2.0094
$OC(CF_3)_3$	1.4437	1.4291	1.7068	1.8531	2.0165
OC_6F_5	1.4759	1.5135	1.7539	1.9036	2.0567

Table S15. Cartesian coordinates for X'Y' (X' = $OTeF_5$, F, Cl, Br, I; Y' = $OTeF_5$, OSF_5, $OSeF_5$, OCF_3, $OC(CF_3)_3$, OC_6F_5) systems optimized at the B3LYP-D3BJ/def2-SVP level of theory.

$(OTeF_5)_2$

Te	-2.094205	0.001466	-0.000220
Te	2.094204	-0.001465	-0.000220
O	-0.483231	-0.519842	-1.009985
O	0.483224	0.520002	-1.009898
F	-3.656819	0.424168	0.880862
F	-1.118504	0.945879	1.265009
F	-2.267054	1.549400	-1.006199
F	-3.050347	-0.955644	-1.265262
F	-1.952763	-1.536822	1.024636
F	3.656838	-0.424313	0.880755
F	1.952820	1.536678	1.024863
F	3.050317	0.955810	-1.265158
F	2.267007	-1.549265	-1.006413
F	1.118520	-0.946039	1.264903

$OTeF_5OSF_5$

Te	-1.578035	-0.000597	0.000298
O	0.058743	0.985943	0.480453
F	-3.188163	-0.797470	-0.417636
F	-0.699503	-1.610525	-0.280841
F	-1.845772	-0.472581	1.774986
F	-2.439266	1.617758	0.272364
F	-1.364551	0.432505	-1.792588
S	2.416659	0.001089	-0.004277
O	1.052175	0.136901	1.015130
F	3.720612	-0.158292	-0.871988
F	2.343354	-1.568545	0.178783
F	3.288074	0.100680	1.308971
F	2.488883	1.569821	-0.184679
F	1.530105	-0.109918	-1.310900

$FOSF_5$

S	-0.284108	-0.005804	0.000003
O	1.181318	0.861930	-0.000005
F	-1.684146	-0.728565	-0.000023
F	0.230396	-0.992668	-1.119244
F	0.230401	-0.992709	1.119213
F	-0.795628	0.998550	1.111378
F	-0.795627	0.998592	-1.111330
F	2.269626	-0.039042	0.000004

$ClOSF_5$

S	-0.553042	0.001592	0.000001
O	0.888882	0.851755	0.000011
F	-1.961442	-0.719244	-0.000018
F	-0.059492	-1.004011	-1.123320
F	-0.059494	-1.004059	1.123278
F	-1.075473	0.996210	1.115871
F	-1.075476	0.996259	-1.115824
Cl	2.342353	-0.013288	0.000001

$BrOSF_5$

S	-1.001976	0.005833	-0.000001
O	0.411875	0.860618	0.000012
F	-2.403046	-0.736938	-0.000013
F	-0.502638	-1.001254	-1.125034
F	-0.502620	-1.001336	1.124948
F	-1.546583	0.988231	1.118194
F	-1.546609	0.988322	-1.118105
Br	2.035717	-0.003186	0.000001

$IOSF_5$

S	-1.374372	0.013051	-0.000005
O	0.019422	0.846153	0.000026
F	-2.775264	-0.740600	-0.000014
F	-0.879675	-1.004548	-1.127665
F	-0.879635	-1.004689	1.127507
F	-1.934276	0.985344	1.121744
F	-1.934329	0.985507	-1.121588
I	1.838928	0.000619	0.000001

$OTeF_5OSeF_5$

Te	-1.845474	-0.001300	-0.000749
O	-0.220870	0.727683	0.855733
F	-3.433074	-0.589933	-0.729302
F	-0.920275	-1.315297	-0.926750
F	-2.085328	-1.183738	1.408451
F	-2.746651	1.325627	0.926157
F	-1.645747	1.161254	-1.432135
Se	2.232127	0.001531	-0.001401
O	0.757294	-0.262636	1.062960
F	3.650872	0.192147	-0.921128

F	2.122433	-1.627167	-0.523011
F	3.160096	-0.507711	1.342681
F	2.357768	1.633178	0.508396
F	1.293342	0.499996	-1.349246

$FOSeF_5$

Se	-0.235838	0.003648	0.000000
F	-1.757978	0.765937	-0.000110
F	0.302915	1.084342	1.211897
F	-0.783001	-1.088090	-1.201117
F	0.302745	1.083734	-1.212517
F	-0.782871	-1.087448	1.201764
O	1.353350	-0.906210	0.000059
F	2.406156	0.033263	0.000028

$ClOSeF_5$

Se	-0.469528	-0.002053	-0.000003
F	-1.999846	0.754355	0.000146
F	0.044865	1.098367	1.215672
F	-1.024745	-1.084882	-1.206620
F	0.044793	1.098542	-1.215584
F	-1.024353	-1.085039	1.206632
O	1.098241	-0.896322	-0.000260
Cl	2.518330	0.012252	-0.000002

$BrOSeF_5$

Se	-0.879069	0.004956	-0.000005
F	-2.400812	-0.774023	0.000062
F	-0.357797	-1.095359	-1.216615
F	-1.456040	1.075165	1.208608
F	-0.357629	-1.095420	1.216430
F	-1.456242	1.075372	-1.208385
O	0.661551	0.909337	-0.000087
Br	2.252932	-0.003281	-0.000001

$IOSeF_5$

Se	-1.232742	0.010448	-0.000021
F	-2.749358	-0.786203	-0.000665
F	-0.711968	-1.093863	-1.220052
F	-1.829718	1.065601	1.213136
F	-0.710976	-1.098013	1.215808
F	-1.830650	1.069915	-1.208939
O	0.286966	0.899977	0.000782
I	2.077576	0.000529	0.000016

$OTeF_5OCF_3$

Te	1.035115	-0.000788	-0.002409
O	-0.639582	0.619336	-0.820768
F	2.680740	-0.477178	0.681300
F	0.224922	-1.331022	1.004496
F	1.292912	-1.217946	-1.378102
F	1.831418	1.341040	-1.000904
F	0.827908	1.189170	1.406697
C	-2.671214	-0.000449	0.001215
O	-1.633704	-0.407981	-0.837409
F	-3.217339	1.135843	-0.412092
F	-3.554898	-0.982031	-0.070709
F	-2.264817	0.159103	1.256359

$FOCF_3$

C	-0.380853	0.006766	0.000012
O	0.721947	-0.844282	0.000160
F	-0.415888	0.779671	1.077342
F	-0.415889	0.779236	-1.077633
F	-1.412358	-0.826308	0.000148
F	1.856306	0.013364	-0.000007

$ClOCF_3$

C	-0.727130	0.006209	-0.000001
O	0.369216	0.839606	-0.000006
F	-0.774946	-0.777413	-1.079056
F	-0.774984	-0.777332	1.079112
F	-1.778520	0.817252	-0.000049
Cl	1.845006	-0.006863	-0.000000

$BrOCF_3$

C	-1.225944	0.011751	0.000000
O	-0.148176	0.851845	-0.000004
F	-1.269599	-0.778170	-1.079631
F	-1.269648	-0.778081	1.079693
F	-2.297514	0.800787	-0.000057
Br	1.487769	-0.002460	-0.000000

$IOCF_3$

C	-1.608577	0.021587	-0.000001
O	-0.538125	0.846758	-0.000024
F	-1.656376	-0.777477	-1.080702
F	-1.656405	-0.777369	1.080776
F	-2.696056	0.793647	-0.000052
I	1.283698	-0.000996	0.000000

$OTeF_5OC(CF_3)_3$

Te	2.230429	0.002171	-0.096997
O	0.485723	0.860362	-0.308244
F	3.927975	-0.699117	0.075520
F	1.512906	-1.615537	0.456076
F	2.191791	-0.591765	-1.852308
F	2.953681	1.619490	-0.635626
F	2.314990	0.580212	1.662489
C	-1.679387	0.001287	-0.079701
O	-0.474711	-0.092912	-0.811224
C	-2.158772	1.492965	0.062637
F	-1.847961	2.183001	-1.031671
F	-3.483559	1.541201	0.236006
F	-1.584501	2.084114	1.112657
C	-2.667164	-0.795401	-1.014684
F	-3.782569	-1.112714	-0.358320
F	-2.990823	-0.057988	-2.077551
F	-2.094126	-1.917245	-1.445958
C	-1.574772	-0.693510	1.320658
F	-1.441213	-2.013164	1.174531
F	-0.516774	-0.245848	1.999630
F	-2.669800	-0.452918	2.047429

$FOC(CF_3)_3$

C	0.001191	-0.007668	-0.264828
O	-0.172216	0.021086	-1.663384
C	-0.903866	1.052835	0.462987
C	-0.239194	-1.449944	0.300171

C	1.519736	0.395872	-0.130840
F	-1.017616	2.150394	-0.279100
F	-2.124461	0.565002	0.691103
F	-0.367483	1.387673	1.642037
F	-1.397797	-1.944769	-0.128935
F	0.734205	-2.270933	-0.106882
F	-0.251897	-1.436644	1.637285
F	1.980963	0.080053	1.078206
F	1.667775	1.707639	-0.316991
F	2.251816	-0.238122	-1.045543
F	-1.574335	-0.013098	-1.937611

ClOC(CF3)3

C	0.055619	-0.007692	-0.152518
O	-0.686637	0.011651	-1.347995
C	-0.419172	1.067218	0.892144
C	0.072527	-1.439111	0.487519
C	1.496729	0.367738	-0.672981
F	-0.821434	2.172513	0.267097
F	-1.436981	0.606668	1.627239
F	0.574155	1.389937	1.726484
F	-1.165066	-1.916669	0.628311
F	0.749146	-2.286581	-0.293161
F	0.652199	-1.417858	1.692658
F	2.424145	0.075134	0.241034
F	1.561581	1.674158	-0.941019
F	1.782490	-0.297579	-1.789358
Cl	-2.389603	-0.001154	-1.233799

BrOC(CF3)3

C	0.365706	-0.007521	-0.096066
O	-0.674437	0.009613	-1.032066
C	0.210969	1.072551	1.036213
C	0.559962	-1.434883	0.524157
C	1.611120	0.358196	-0.995506
F	-0.345008	2.179677	0.544620
F	-0.568193	0.619956	2.026395
F	1.396227	1.392735	1.563676
F	-0.592984	-1.906449	1.005819
F	0.989994	-2.290719	-0.407467
F	1.450205	-1.412778	1.522134
F	2.755054	0.068608	-0.370125
F	1.602665	1.662881	-1.281082
F	1.578829	-0.315617	-2.142132
Br	-2.442633	0.000240	-0.477508

IOC(CF3)3

C	0.686534	-0.006574	-0.088514
O	-0.432713	0.007923	-0.912427
C	0.638010	1.072250	1.053521
C	0.934652	-1.431670	0.517469
C	1.853132	0.354260	-1.088746
F	0.049937	2.184924	0.610485
F	-0.068518	0.624449	2.101697
F	1.860926	1.385662	1.489135
F	-0.179688	-1.899231	1.092297
F	1.283862	-2.292891	-0.442378
F	1.901243	-1.414041	1.441603
F	3.048193	0.068895	-0.561657
F	1.821176	1.658493	-1.379354
F	1.726189	-0.324190	-2.225957
I	-2.343436	0.001479	-0.267845

OTeF5OC6F5

Te	2.158769	0.010335	0.169354
O	0.798207	1.201823	-0.561247
F	3.450441	-1.050232	0.956132
F	0.861981	-1.002488	1.052131
F	2.094478	-1.162942	-1.267568
F	3.504798	0.972449	-0.674751
F	2.256179	1.173581	1.616603
O	-0.134754	0.517985	-1.477956
C	-1.304008	0.275284	-0.824458
C	-1.705552	-1.044868	-0.611735
C	-2.142437	1.320230	-0.427900
C	-2.932354	-1.317456	-0.026439
C	-3.357654	1.050808	0.177201
C	-3.751070	-0.269266	0.374115
F	-0.927015	-2.047324	-0.990565
F	-3.323986	-2.574058	0.161952
F	-4.920894	-0.527426	0.942788
F	-4.157030	2.042731	0.558667
F	-1.772858	2.577459	-0.628442

FOC6F5

C	-1.112892	0.000052	-0.259255
C	-0.407105	-1.209513	-0.160950
C	0.967706	-1.210937	-0.007606
C	1.649311	-0.000059	0.070760
C	0.967792	1.210885	-0.007517
C	-0.406984	1.209575	-0.161074
O	-2.429012	0.000099	-0.480535
F	-1.057391	-2.358568	-0.227485
F	1.644025	-2.353962	0.067254
F	2.965868	-0.000106	0.214892
F	1.644234	2.353842	0.067291
F	-1.057210	2.358671	-0.227473
F	-3.085625	0.000032	0.883092

ClOC6F5

C	-0.800073	0.000104	-0.392465
C	-0.104745	-1.202665	-0.249224
C	1.257835	-1.205972	0.002616
C	1.937138	-0.000073	0.128407
C	1.258086	1.205902	0.002685
C	-0.104562	1.202703	-0.249142
O	-2.112830	0.000313	-0.730555
F	-0.752756	-2.356303	-0.358564
F	1.919815	-2.354780	0.124142
F	3.243991	-0.000293	0.365968
F	1.920082	2.354666	0.124202
F	-0.752355	2.356492	-0.358475
Cl	-3.174613	-0.000031	0.665394

BrOC6F5

C	-0.267502	-0.000206	0.534380
C	0.420247	-1.202311	0.337480
C	1.760123	-1.205702	-0.013558
C	2.428841	0.000213	-0.188404
C	1.759729	1.205863	-0.013507
C	0.419826	1.202106	0.337626
O	-1.542421	-0.000587	0.969382

F	-0.218828	-2.356772	0.490383
F	2.411634	-2.354602	-0.183861
F	3.715186	0.000467	-0.520388
F	2.410924	2.354968	-0.183643
F	-0.219458	2.356416	0.490593
Br	-2.848095	0.000018	-0.415912

IOC_6F_5

C	0.178550	0.000356	0.598827
C	0.861817	-1.199988	0.375857
C	2.187685	-1.204539	-0.025195
C	2.851682	-0.000255	-0.224887
C	2.188273	1.204229	-0.025383
C	0.862253	1.200202	0.375713
O	-1.077735	0.000711	1.077612
F	0.226994	-2.356327	0.550191
F	2.830567	-2.355267	-0.219690
F	4.126271	-0.000687	-0.604160
F	2.831329	2.354793	-0.219963
F	0.228105	2.356951	0.549809
I	-2.610360	-0.000017	-0.293890

QTAIM charges of X'Y' systems

Table S16. QTAIM charges (in $|e^-|$) for X'–Y' systems with X' = Br and I.

Y' =	X' = Br	X' = I
	q(Y')	*q*(Y')
OSF_5	-0.40	-0.55
$OSeF_5$	-0.41	-0.56
OCF_3	-0.38	-0.52
$OC(CF_3)_3$	-0.37	-0.52
OC_6F_5	-0.26	-0.41

EDFs for X'Y' systems

Table S17. EDF probabilities for three-basin EDFs analysis of X'Y' when X' = $OTeF_5$.

RSRS	Y' =					
	$OTeF_5$	OSF_5	$OSeF_5$	OCF_3	$OC(CF_3)_3$	OC_6F_5
$[OTeF_5]^0[Y']^0$	0.429	0.429	0.426	0.439	0.431	0.432
$[OTeF_5]^-[Y']^+$	0.248	0.249	0.245	0.252	0.253	0.278
$[OTeF_5]^+[Y']^-$	0.248	0.247	0.249	0.244	0.243	0.218
$[OTeF_5]^{2-}[Y']^{2+}$	0.035	0.036	0.036	0.032	0.036	0.042
$[OTeF_5]^{2+}[Y']^{2-}$	0.035	0.035	0.038	0.029	0.033	0.025
$[OTeF_5]^{3-}[Y']^{3+}$	0.002	0.002	0.002	0.002	0.002	0.003
$[OTeF_5]^{3+}[Y']^{3-}$	0.002	0.002	0.003	0.002	0.002	0.001

Table S18. EDF probabilities for three-basin EDFs analysis of X'Y' when X' = F.

RSRS	Y' =					
	$OTeF_5$	OSF_5	$OSeF_5$	OCF_3	$OC(CF_3)_3$	OC_6F_5
$[F]^0[Y']^0$	0.445	0.446	0.445	0.449	0.443	0.443
$[F]^-[Y']^+$	0.310	0.309	0.307	0.314	0.315	0.349
$[F]^+[Y']^-$	0.195	0.196	0.197	0.192	0.191	0.166
$[F]^{2-}[Y']^{2+}$	0.029	0.029	0.029	0.027	0.031	0.030
$[F]^{2+}[Y']^{2-}$	0.019	0.019	0.019	0.016	0.018	0.012
$[F]^{3-}[Y']^{3+}$	0.001	0.001	0.001	0.001	0.001	0.001
$[F]^{3+}[Y']^{3-}$	0.000	0.001	0.001	0.001	0.001	0.000

Table S19. EDF probabilities for three-basin EDFs analysis of X'Y' when X' = Cl.

RSRS	Y' =					
	$OTeF_5$	OSF_5	$OSeF_5$	OCF_3	$OC(CF_3)_3$	OC_6F_5
$[Cl]^0[Y']^0$	0.412	0.414	0.411	0.423	0.418	0.440
$[Cl]^-[Y']^0$	0.151	0.151	0.150	0.156	0.160	0.186
$[Cl]^+[Y']^-$	0.364	0.365	0.365	0.360	0.352	0.321
$[Cl]^{2-}[Y']^{2+}$	0.016	0.015	0.015	0.014	0.017	0.018
$[Cl]^{2+}[Y']^{2-}$	0.054	0.051	0.055	0.043	0.049	0.033
$[Cl]^{3-}[Y']^{3+}$	0.000	0.001	0.001	0.001	0.001	0.001
$[Cl]^{3+}[Y']^{3-}$	0.003	0.003	0.003	0.002	0.003	0.001

Table S20. EDF probabilities for three-basin EDFs analysis of X'Y' when X' = Br.

RSRS	Y' =					
	$OTeF_5$	OSF_5	$OSeF_5$	OCF_3	$OC(CF_3)_3$	OC_6F_5
$[Br]^0[Y']^0$	0.391	0.393	0.391	0.403	0.399	0.430
$[Br]^-[Y']^0$	0.109	0.119	0.118	0.123	0.129	0.157
$[Br]^+[Y']^-$	0.421	0.416	0.415	0.411	0.399	0.360
$[Br]^{2-}[Y']^{2+}$	0.015	0.010	0.010	0.010	0.012	0.013
$[Br]^{2+}[Y']^{2-}$	0.058	0.058	0.062	0.050	0.057	0.038
$[Br]^{3-}[Y']^{3+}$	0.000	0.000	0.000	0.000	0.001	0.001
$[Br]^{3+}[Y']^{3-}$	0.001	0.003	0.004	0.003	0.004	0.002

Table S21. EDF probabilities for three-basin EDFs analysis of X'Y' when X' = I.

RSRS	Y' =					
	$OTeF_5$	OSF_5	$OSeF_5$	OCF_3	$OC(CF_3)_3$	OC_6F_5
$[I]^0[Y']^0$	0.341	0.349	0.348	0.361	0.358	0.394
$[I]^-[Y']^0$	0.078	0.081	0.082	0.086	0.091	0.113
$[I]^+[Y']^-$	0.486	0.488	0.484	0.481	0.466	0.432
$[I]^{2-}[Y']^{2+}$	0.005	0.006	0.006	0.006	0.008	0.009
$[I]^{2+}[Y']^{2-}$	0.083	0.071	0.075	0.062	0.071	0.049
$[I]^{3+}[Y']^{3-}$	0.006	0.004	0.005	0.003	0.005	0.002

IQA interaction energies and DIs for X'Y' systems

Table S22. IQA interaction terms (values in kcal·mol^{-1}) and delocalization indices for X'Y' when X' = $OTeF_5$.

Y' =	X' = $OTeF_5$			
	E_{int}	V_{cl}	V_{xc}	DI
$OTeF_5$	-222.0	34.1	-256.1	1.65
OSF_5	-224.4	35.6	-260.1	1.65
$OSeF_5$	-225.5	36.9	-262.4	1.68
OCF_3	-212.8	32.9	-245.8	1.55
$OC(CF_3)_3$	-221.6	29.1	-250.8	1.63
OC_6F_5	-207.2	23.7	-230.9	1.59

Table S23. IQA interaction terms (values in kcal·mol^{-1}) and delocalization indices for X'Y' when X' = F.

Y' =	X' = F			
	E_{int}	V_{cl}	V_{xc}	DI
OSF_5	-203.5	24.4	-227.9	1.39
$OSeF_5$	-203.4	24.8	-228.1	1.40
OCF_3	-198.5	22.1	-220.6	1.35
$OC(CF_3)_3$	-205.5	18.6	-224.1	1.40
OC_6F_5	-179.0	6.0	-185.0	1.29

Table S24. IQA interaction terms (values in kcal·mol^{-1}) and delocalization indices for X'Y' when X' = Cl.

Y' =	X' = Cl			
	E_{int}	V_{cl}	V_{xc}	DI
OSF_5	-222.3	-12.4	-209.9	1.46
$OSeF_5$	-223.5	-11.4	-212.1	1.48
OCF_3	-213.2	-9.6	-203.6	1.41
$OC(CF_3)_3$	-219.1	-8.6	-210.5	1.49
OC_6F_5	-190.3	-1.2	-189.2	1.41

Table S25. IQA interaction terms (values in kcal·mol^{-1}) and delocalization indices for X'Y' when X' = Br.

Y' =	X' = Br			
	E_{int}	V_{cl}	V_{xc}	DI
OSF_5	-222.7	-43.8	-178.8	1.36
$OSeF_5$	-223.3	-42.6	-180.7	1.39
OCF_3	-215.5	-40.5	-174.9	1.32
$OC(CF_3)_3$	-220.6	-38.5	-182.0	1.41
OC_6F_5	-186.8	-23.1	-163.8	1.36

Table S26. IQA interaction terms (values in kcal·mol^{-1}) and delocalization indices for X'Y' when X' = I.

Y' =	X' = I			
	E_{int}	V_{cl}	V_{xc}	DI
OSF_5	-236.6	-87.1	-149.5	1.24
$OSeF_5$	-236.1	-84.9	-151.2	1.26
OCF_3	-230.7	-83.0	-147.7	1.21
$OC(CF_3)_3$	-237.3	-82.0	-155.3	1.31
OC_6F_5	-204.7	-62.0	-142.7	1.28
